# High Particle Number Emissions Determined with Robust Regression Plume Analysis (RRPA) from Hundreds of Vehicle Chases

**DOI:** 10.1021/acs.est.2c08198

**Published:** 2023-06-07

**Authors:** Miska Olin, Henri Oikarinen, Petteri Marjanen, Santtu Mikkonen, Panu Karjalainen

**Affiliations:** † Aerosol Physics Laboratory, Tampere University, FI-33014 Tampere, Finland; ‡ Department of Technical Physics, University of Eastern Finland, FI-70211 Kuopio, Finland; § Department of Environmental and Biological Sciences, University of Eastern Finland, FI-70211 Kuopio, Finland; ∥ Institute for Advanced Study, Tampere University, FI-33014 Tampere, Finland

**Keywords:** passenger cars, real-world emissions, emission factors, particle emissions, particle number, vehicle fleet, RRPA

## Abstract

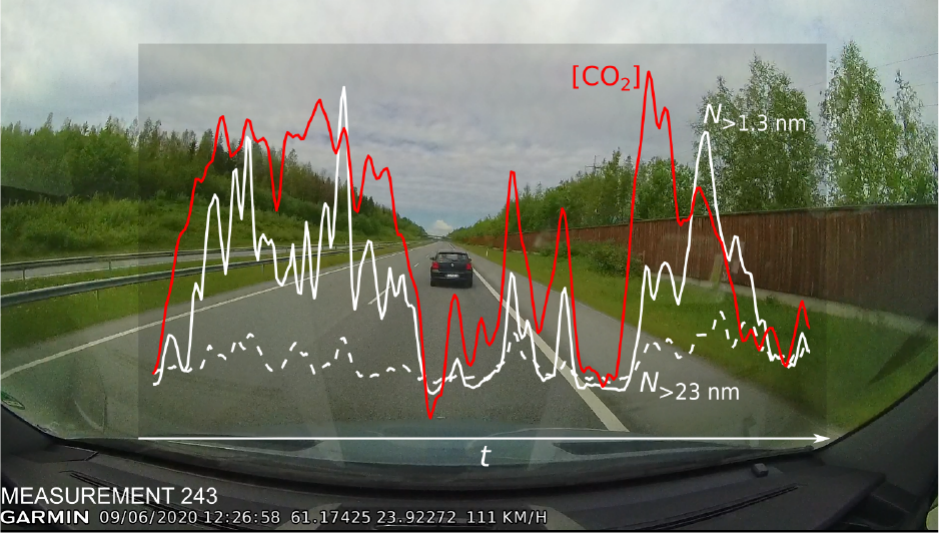

Particle number emission factors were determined for hundreds of individual diesel and gasoline vehicles in their real operation on Finnish highways and regional roads in 2020 with one-by-one chase measurements and Robust Regression Plume Analysis (RRPA). RRPA is a rapid way to analyze data from a large number of vehicle chases automatically. The particle number emission factors were determined for four ranges of particle diameters (>1.3, > 2.5, > 10, and >23 nm). The emission factors for most of the measured vehicles were observed to significantly exceed the non-volatile particle number limits used in the most recent European emission regulation levels, for both light-duty and heavy-duty vehicles. Additionally, most of the newest vehicles (covering regulation levels up to Euro 6), for which the particle number emission regulations (non-volatile >23 nm particles) apply, showed emission factors of the >23 nm particles clearly above the regulation limits. Although the experiments included measurements of real-world plume particles (mixture of non-volatile and semi-volatile particles) and not only the non-volatile regulated particles, it is important to note that the emissions of regulated particles were also estimated to exceed the limits, based on non-volatile >23 nm particle fraction from curbside studies. Moreover, the emission factors of the >1.3 nm particles were mostly about an order of magnitude higher compared to the >23 nm particles.

## Abstract 2

Particle number emission factors of hundreds of individual vehicles measured in their real operation exceed the particle number limits in European emission regulations by orders of magnitude.

## Introduction

Road traffic is one of the main sources of emissions worsening air quality and being detrimental to human health. The highest particle concentrations have been observed at locations with dense traffic, such as on highways, on streets (e.g., Kittelson et al.^1^), and in street canyons (e.g., Wehner et al.,^2^ Hietikko et al.,^3^ Olin et al.,^4,5^ Lintusaari et al.^6^). Adverse health effects of particles are usually associated with particle mass (PM) concentrations in the ambient air, but the health risks are suggested to be more strongly linked with the particle number (PN) concentration.^7−9^

Some traffic-related emissions, such as PM, total hydrocarbons, carbon monoxide, and nitrogen oxides, have been regulated in almost every country for newly sold vehicles.^10^ Non-volatile PN emissions have been added to the regulation in Europe and Asia starting with Euro 5b for light-duty (LD) diesel vehicles since the registration year 2013. This PN emission limit (6 × 10^11^/km in chassis dynamometer tests) applies also to passenger cars with gasoline direct injection (GDI) since the Euro 6c level and to heavy-duty (HD) on-road diesel vehicles since the Euro 6 level for which the limit is 8 × 10^11^/kWh. This regulation limit does not consider either semi-volatile particles of any size or sub-23 nm particles of any volatility. However, the existence of both of these particle types is expected. The real-world particle emissions can be orders of magnitude higher than the regulatory limit^11^ because the regulated particle emissions include basically only primary particle emissions, which are particles formed during the combustion and exhaust after treatment processes and are mostly non-volatile. Primary emissions can, however, contain also semi-volatile particles, which can originate from uncombusted lubricant oil in cylinders, which is found to be emitted during engine braking.^12^ The non-volatile particles are selected for controlling the PN emissions due to better repeatability in laboratory settings. After the exhaust has been released from the tailpipe, rapid cooling of the exhaust plume tends to both form new particles via nucleation and to grow the existing and the newly formed ones via condensation with low-volatility vapors.^13^ This add-on to the primary particle emissions, called delayed primary emissions,^14^ typically consists mainly of semi-volatile particles and semi-volatile material in the particles, and it depends greatly on the location and the environmental conditions outdoors and is thus disregarded from the PN regulations.

The breakthrough in the particle emission control of diesel vehicles has been achieved with the diesel particle filters (DPFs) in the majority of diesel applications as a part of the exhaust aftertreatment (EAT) system. DPFs effectively filter the exhaust before it is emitted to the atmosphere and have been shown to reduce real-world PM emissions by at least 90% and PN emissions even more,^15−17^ even down to the detection limits of particle counters. Although the PN emission limit introduced with the Euro 5b level has been a requisite since 2013, DPFs have been applied in many vehicles several years earlier in practice.

PN concentrations in gasoline vehicle exhaust have traditionally been lower compared to diesel vehicles without a DPF.^18^ However, the general need to enhance the fuel economy has led to the use of direct fuel injection techniques also in gasoline vehicles (GDI),^19^ which has led to increased particle emissions of gasoline vehicles.^20−22^ Similarly to diesel vehicles, PN emissions of GDI vehicles are dominated by ultrafine particles.^11,23^

The passenger car fleet in Finland is a mixture of new and old engine and EAT technologies. The average passenger car age is about 12 years, which also represents the European average.^24^ In addition to traditional combustion engine technologies, advanced technologies, such as electric and hybrid vehicles in different forms, have increased their share on the market. The vehicles currently operating on-road have passed the emission standards via laboratory tests when they were new. However, it is largely unknown how their emissions have evolved due to their usage. Emission tests in statutory periodical technical inspections performed for vehicles on idle have historically not covered PN emissions, but they will be covered in some countries in the near future, with The Netherlands and Belgium being in the first front.^25^ It has been known for some years in the scientific community that existing emission tests cannot be used to properly determine real-world emissions of total particles, due to the difference between the formation and evolution of emissions in the laboratory sampling systems and in a real atmosphere.^26^

Particle emissions of vehicle fleets in their real-use environment have previously been determined, e.g., by measuring particle concentrations or distributions at the curbside of a street in many different locations.^3−6,27−32^ Connecting the measured particle variables with carbon dioxide (CO_2_) concentrations^3−6,27,30−32^ provides data for particle emissions per amounts of fuel combusted, i.e., the emission factors (EFs) of particles. Curbside measurements in high-traffic locations can only provide EFs for the whole fleet at a time (fleet average) because distinguishing the emissions from different vehicles is almost impossible. Data measured at curbside locations with less traffic can include individually separable signals from individual vehicles passing by the measurement location. This type of curbside measurements, also called remote-sensing measurements, have been performed, e.g., by Hansen and Rosen,^27^ Ježek et al.,^30^ and Ban-Weiss et al.^33^ for vehicle particle emissions and by Herndon et al.^34^ for aircraft nitrogen oxides emissions. However, these stationary curbside measurements provide only snapshots of the emissions from the vehicles passing by the measurement location, representing only a small limited operation of a vehicle, causing signals of less than a minute and only at specific locations.^35^

Several vehicle emission studies have been performed with a mobile measurement platform chasing individual vehicles both in public traffic^31,36−42^ and in private or quiet public traffic areas.^11−13,17,30,42^ Many of these chase experiments focused on emissions from buses or from HD vehicles^12,13,36−38,40,42^ or considered only gaseous emissions.^37−39^ Chase experiments involving wide sets of vehicles selected randomly on public roads were conducted, e.g., by Ježek et al.,^31^ Zavala et al.,^39^ and Park et al.^41^ Zavala et al.^39^ reported EFs of gaseous pollutants from 345 vehicles driving in the Mexico City Metropolitan Area in 2003; however, they reported emissions from LD vehicles as fleet averages only and they did not report particle emissions. Park et al.^41^ determined PN emission factors of >10 nm particles for 143 LD gasoline and 93 HD diesel vehicles driving in Los Angeles County in 2007 with different driving dynamics. Ježek et al.^31^ reported PN emission factors of >5.6 nm particles for 139 vehicles of different types driving on Slovenian highways and regional roads in 2011. They also categorized the results according to vehicle age and engine power. However, EFs of particles for more recent vehicle fleets, e.g., covering also Euro 6 level vehicles, have not been determined with chase measurements lately. Novel findings^14^ have indicated that traffic can also be a major source of nanocluster aerosol (NCA), i.e., particles sized between ∼1 and ∼3 nm. The exact formation mechanisms of these particles are not yet well-known, and the NCA emissions overall are not yet very thoroughly quantified with respect to vehicle type, technology, and age.

In this study, PN emission factors beginning from the particle diameter of 1.3 nm were determined for 253 randomly selected vehicles driving on public Finnish highways and regional roads with one-by-one chase measurements in 2020, providing a good coverage of EFs for Euro 6 level vehicles as well. Additionally, specific attention was paid on the method to analyze the data from a large number of vehicle chases efficiently. The data from the chase measurements were analyzed via Robust Regression Plume Analysis (RRPA), a method developed in this study.

## Materials and Methods

### Measurement Setup

**Figure 1:**
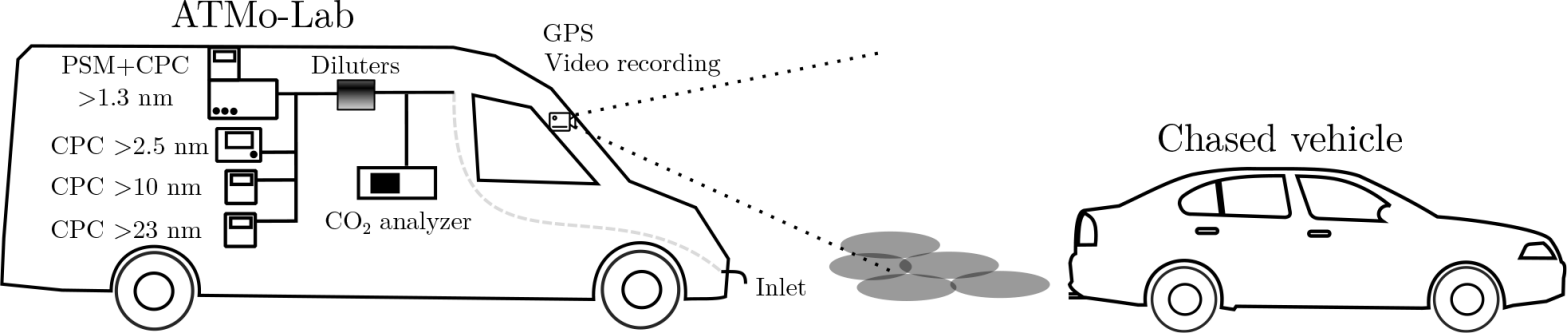
Measurement setup installed in the measurement van, ATMo-Lab, and an example of a vehicle to be chased.

Measurement devices were installed into the Tampere University’s measurement van, the Aerosol and Trace Gas Mobile Laboratory (ATMo-Lab^43^), as shown in Figure 1. The sample was drawn through a downward-pointing inlet near the front bumper using the suction of the measurement devices (30 slpm). The total length of the sampling line was 4.5 m, and its inner diameter varied between 4 and 9 mm depending on the location inside the van (detailed information on the sampling system and biases in particle measurement caused by that can be found in the following sections and in the Supporting Information Text S1.1). The measurement data from the devices shown in Figure 1 were utilized in this study; additionally, there were also some other devices attached to the same sampling lines but that data are not presented in this paper.

The CO_2_ concentration ([CO_2_]) was measured with a LI-COR CO_2_/H_2_O gas analyzer. The PN concentration (*N*) was measured with four condensation particle counters (CPCs) with a variety of cutoff particle diameters (1.3, 2.5, 10, and 23 nm). The lowest cutoff of 1.3 nm was achieved using the combination of an Airmodus Particle Size Magnifier (PSM A10) and an Airmodus CPC A20. The CPC having the cutoff of 2.5 nm was a TSI CPC 3756, and the rest were Airmodus CPCs A20 (10 nm) and A23 (23 nm). This set of different cutoff diameters provides information on the particle size distributions in four size bins (1.3–2.5, 2.5–10, 10–23, and >23 nm).

The particle concentrations in the sample were diluted using a set of two customized bifurcated flow diluters (see details from the Supporting Information Text S1) in order to keep the concentrations within a suitable range for the particle counters. The concentrations of > 23 nm particles were mostly within the range 10^3^–10^5^ cm^–3^ and of >1.3 nm particles within the range 10^3^–10^6^ cm^–3^, whereas the upper limits of the CPCs are about 10^5^ cm^–3^, which would have been exceeded 6–10% of the time (depending on the CPC model) without a diluter set. The dilution ratio (DR) of this diluter set for >50 nm particles was measured to be 98 ± 4 by using a test dioctyl sebacate aerosol during the chase experiments.

### Measurement Location and Protocol

The chase measurements were conducted during 17 days in June 2020 near surrounding roads of Tampere, Finland. The Tampere region is the second largest metropolitan area in Finland with ∼400,000 inhabitants. Outdoor temperatures were within the range 15–30 °C, and there was almost no precipitation during the measurements.

In total, 353 vehicles were chased, of which only the most successful ones in terms of capturing the exhaust plume of the studied vehicles properly and of having most of the measurement devices functioning properly (253 chases) were taken into account in data analysis. Most of the chases were conducted on highways with two lanes in one direction or on regional roads having the speed limits of 100 or 120 km/h. Twenty-two of the 253 chases were conducted on regional roads having the speed limits of 60 to 80 km/h. Urban areas were avoided due to mixing of the exhaust plumes from several individual vehicles with higher traffic densities. The measurements were conducted during a day while avoiding morning and afternoon rush hours to minimize the disturbance of plumes from other traffic.

The chase distance was kept as safe and constant but as short as possible; it ranged between 5 and 50 m, depending on the driving velocity. According to Rönkkö et al.,^13^ 5 m is enough for the delayed primary particle emissions to fully form behind an emitting vehicle driving on road. A single chase lasted between 1 and 8 min; the mean value of all chases was 4 min. Specific technical details of the vehicles were obtained from the license plates using the Traficom database. The chased vehicles were randomly selected from the public traffic, and the drivers were mostly unaware of the measurement. Vehicles clearly exceeding the speed limits were not chased; thus, the data can be slightly biased by excluding this activity.

### Determining Emission Factors from Chase Events with Robust Regression Plume Analysis (RRPA)

The measurement data were first processed by time-synchronizing the data from different devices due to different sample residence times in the sampling lines. In addition, the data was corrected for diffusional losses of particles onto the sampling lines (both straight and bent parts) and in the bifurcated flow diluter set, for the DR of the recorded particle concentrations, and for the maximum detection efficiencies of the CPCs. Biases in measurement of large particles, such as caused by aspiration of the inlet and inertial losses in the bends, are not corrected because the focus of this study is on smaller particles. Due to an approximately 3 s uncertainty involved in the time synchronization, the measured 1 s data were averaged to 3 s to improve the accuracy of the results. Detailed information on data processing can be found in the Supporting Information Text S1.

Emission factors of chase events (chase measurements of single vehicles) were analyzed using Robust Regression Plume Analysis (RRPA) developed here. It provides an efficient way to analyze plume data without the need to determine background concentrations. Determining the background concentrations usually requires a lot of manual work, such as screening the time series for searching the time ranges that most properly represent the background signals. Additionally, partly due to this manual searching, determining the background concentrations usually involves high uncertainty (see the Supporting Information Text S2), especially with more recent vehicle technologies emitting smaller amounts of both CO_2_ and other pollutants and thus causing smaller increases in the detected concentrations between the background signal and the chase event. The background concentrations are needed in the integration method (eq S13), which has been used in the majority of vehicle exhaust studies with curbside^27,30−32^ or chase^31,36,39−42^ measurements. It can be shown (see the Supporting Information Text S2) that, by using the general assumption that both CO_2_ and aerosol dilute equally in a turbulent exhaust plume between the tailpipe and the sampling inlet^44^ and by assuming a constant EF throughout a chase event, the slope in the *N* vs [CO_2_] plot substituted in eq S18 (the slope method) gives the EF in the unit of emitted particles per emitted CO_2_ mass even without explicitly knowing the background concentrations of particles and CO_2_. This is the main concept in RRPA. Slope methods of similar types to the one in this study have been used, e.g., by Herndon et al.,^34^ Canagaratna et al.,^36^ Herndon et al.,^37^ Shorter et al.,^38^ and Zavala et al.,^39^ of which Canagaratna et al.^36^ reported EFs for particles as well. They determined particle mass EFs for hundreds of HD vehicle chase events using both an integration and a slope method and concluded that the methods agree to within 15% with no systematic deviation. See the Supporting Information Text S2 for the detailed description and comparison of the slope and integration methods used here.

Another key concept in RRPA is the automatic downweighting of the data points that may have been disturbed, for example, by other traffic in the vicinity of the chase measurements. This is done by using a robust regression method in fitting the slopes in the *N* vs [CO_2_] plots. The selected robust regression method uses an iteratively reweighted least-squares algorithm, which sets the weights for all data points used in linear fitting, using bisquare weighting.^45^ The amount of manual work is further minimized with robust regression, because manual time series screening is not needed in excluding the disturbed data points, using, for example, recorded videos from the chase measurements.

**Figure 2:**
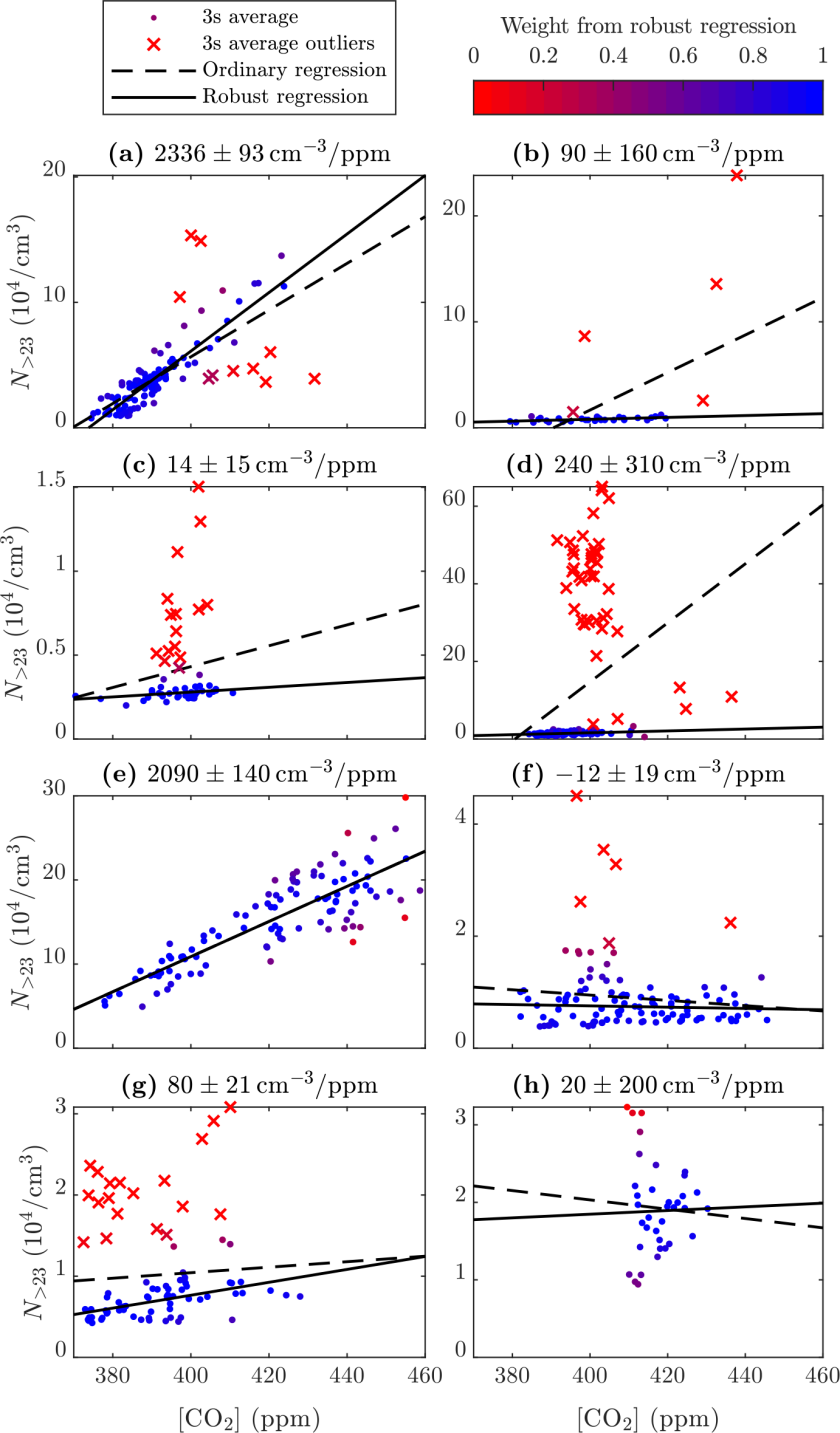
Example data of the number concentration of particles larger than 23 nm (*N*_>23_) versus carbon dioxide concentration ([CO_2_]) from eight chase events of eight unique vehicle chases. The slopes and their standard errors from the robust regressions are shown in the titles.

The applicability of RRPA is evaluated next with eight example chase events in Figure 2. The number concentrations of particles larger than 23 nm (*N*_>23_) as functions of [CO_2_] measured in chase events are presented. The data analyzed for a single chase event are from the time range of the chase (start and end time stamps defined by the researchers during the measurements) plus 6 s extensions before the start time and after the end time, in order to include additional signal from the background. For data interpretation purposes, the data points getting the weights of nearly zero upon robust regression are marked as outliers.

The examples in Figure 2 represent different situations and levels of performance of robust regressions. Whereas [CO_2_] in raw exhaust is around 10^5^ ppm, the highest concentrations measured from the plumes are only less than 500 ppm while the background concentration was about 370 ppm. This denotes that the exhausts have been diluted with the DRs around 10^3^ at the chase distances. Despite these quite high DRs, RRPA seems to function properly in most cases. Figure 2a,b represents cases where the robust regression successfully removes small numbers of outliers; especially, the fitting in Figure 2b benefits greatly from the robust regression because the slope from the ordinary regression is much higher, only because of the small number of data points having notably high *N*_>23_ values. This type of outliers can be originated, for example, from overtaking vehicles. Oncoming vehicles are expected to cause fewer disturbances in the data from highway driving—as is the case in Figure 2a,b—due to the wide area between the lanes to the opposite directions resulting in strong dilution. According to the recorded videos, the outliers in Figure 2a originate from times when the chased vehicle overtook two trucks, of which exhaust plumes are presumably the causes for the outliers. The outliers in Figure 2b originate from another vehicle overtaking the chased vehicle and from the chased vehicle accelerating during the very last seconds of the chase. This implies that the end time stamp of the chase should have been marked some seconds earlier; nevertheless, the robust regression is capable in correcting this type of an error.

Figure 2c,d represents cases where the robust regression performs well even with many outliers. This type of outliers, in turn, may have originated from the change of the surrounding area, because there seems to be clusters of different *N*_>23_ levels with almost constant [CO_2_] levels. This can also be observed from the recorded videos in the case of Figure 2c; however, the number of outliers in the case of Figure 2d is large partially due to the chased vehicle overtaking another vehicle very slowly (taking nearly 1 min), which may also be interpreted so that the surrounding area became to include the overtaken vehicle.

Figure 2e represents a case without any clear outliers, and the robust and ordinary regressions result in similar linear fits. Figure 2f shows an example data resulting in a negative slope. However, it has a high relative uncertainty (standard error normalized with the absolute value of the slope), which denotes that the EF can also be positive but is anyway very low. Relative uncertainties are typically high in cases with low EFs because the particle concentrations in the plume are so close to the background concentrations. Figure 2g represents a relatively rare example where the robust regression cannot be considered very reliable because there are two clusters of data points and it considers the upper ones the outliers. In reality, the upper ones could still be the actual chase event data; nevertheless, this is not expected in this case since there are more data points in the lower cluster. The ordinary regression, in turn, results in a very low EF. According to the videos, the upper cluster is caused by several other vehicles disturbing the chase. Figure 2h represents data without any clear linear behavior, regardless of the chosen regression method. In this case, the relative uncertainty becomes very high. No clear explanation for this cannot be found from the videos; one possibility is that capturing the plume did not vary enough (narrow [CO_2_] range). The range of measured [CO_2_] levels (from the background level to the highest level in the plume) during a measurement describes how well the exhaust plume was captured and varied, which is desired in the slope method for revealing the linear behavior. A too narrow [CO_2_] range represents a not very successful chase event, for which linear fitting does not result in a very reliable EF. These unsuccessful chase events were neglected from the data analysis by using a criterion that the standard deviation of [CO_2_] during the chase event needs to be higher than 5 ppm. Figure 2e,f represents examples with wide [CO_2_] ranges. The slope in Figure 2e has also a low relative uncertainty, but a high relative uncertainty is involved in the slope in Figure 2f, caused by the low value of the slope. Both measurements (Figure 2e,f) can still be considered very successful—in terms of capturing the plume properly—due to the wide [CO_2_] ranges. In contrast, the data in Figure 2h have a very narrow [CO_2_] range (standard deviation of 5.6 ppm). Table S3 shows a comparison between this slope method with robust regression (RRPA) and the integration method applied in these example chase events.

## Results and Discussion

### Distribution of Emissions Factors

**Figure 3:**
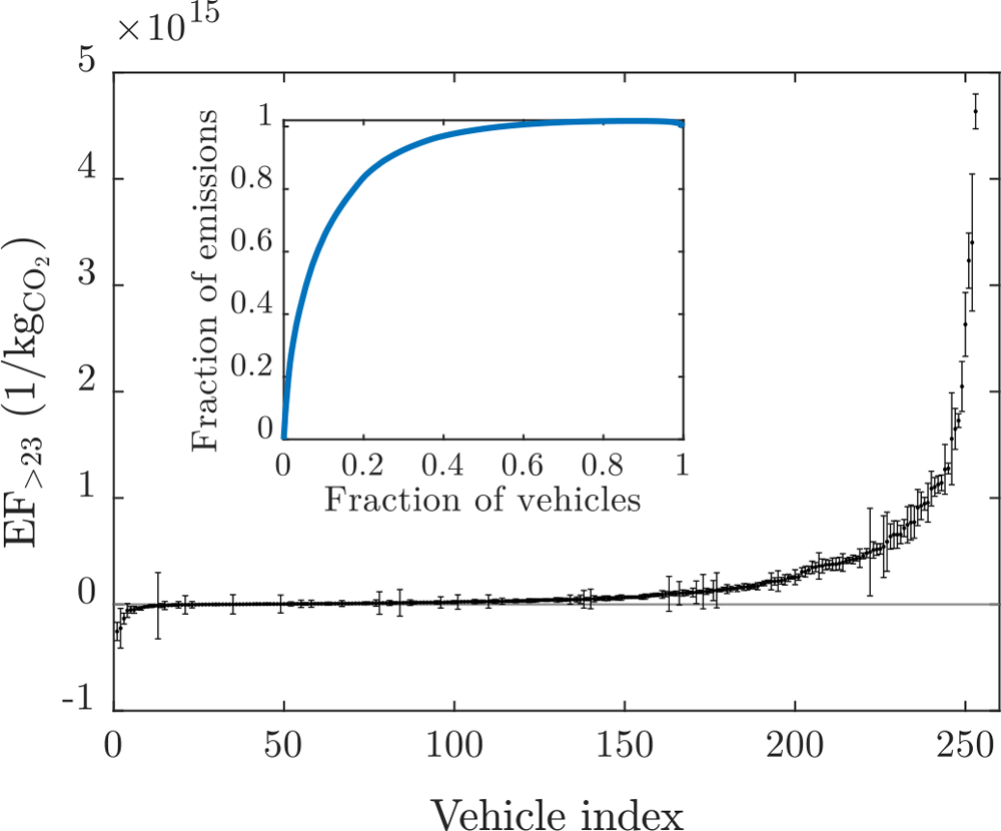
Emission factors of particles larger than 23 nm (EF_>23_) of all vehicles. The vehicles are sorted according to their emission factors. The error bars denote the standard errors of the slopes in the robust regressions. The inserted plot shows the same data cumulatively beginning from the most emitting vehicles (e.g., the most emitting 20% of vehicles contribute to over 80% of >23 nm particle number emissions).

EFs are expressed with four different units in this paper: how many particles are emitted (1) with 1 kg of CO_2_ emitted, (2) with 1 kg of fuel combusted, (3) with 1 km driven, and (4) with 1 kWh of energy produced (see Table S4 and corresponding Text S2.4 of the Supporting Information for details). The results for emission factors of >23 nm particles (EF_>23_) from all valid chase events are presented in Figure 3. Sorting the vehicles according to their EFs provides insight into how the EFs are distributed. The range of EFs is wide, with a small number of vehicles exceeding 10^15^/kg_CO_2__. On the other hand, even negative EFs are observed but are mainly caused by the inaccuracy in fitting the slopes for low-emitters. Using the definition of the EF of this study—which slightly differs from the definitions used elsewhere—an EF can be negative if a vehicle emits no particles while the outdoor air consumed for combustion contains particles (see the Supporting Information Text S2). The most negative EF practically possible is on the order of −10^13^/kg_CO_2__, being an order of magnitude less negative than the most negative EFs observed here. To assess the reliability of using robust regression in this study is examined in Figure S2, which shows what the distribution of EF_>23_ will look like if ordinary regression is used instead. It can be observed that the feature of the distribution as in Figure 3 is not greatly affected by the chosen regression method (compare Figure 3 to Figure S2a). However, EFs for a small number of vehicles were notably different with the ordinary regression (compare Figure 3 to Figure S2b), due to ordinary regression being oversensitive to the included outlying data points (see Mikkonen et al.^46^). The relative uncertainties are also generally lower when using the robust regression.

**Figure 4:**
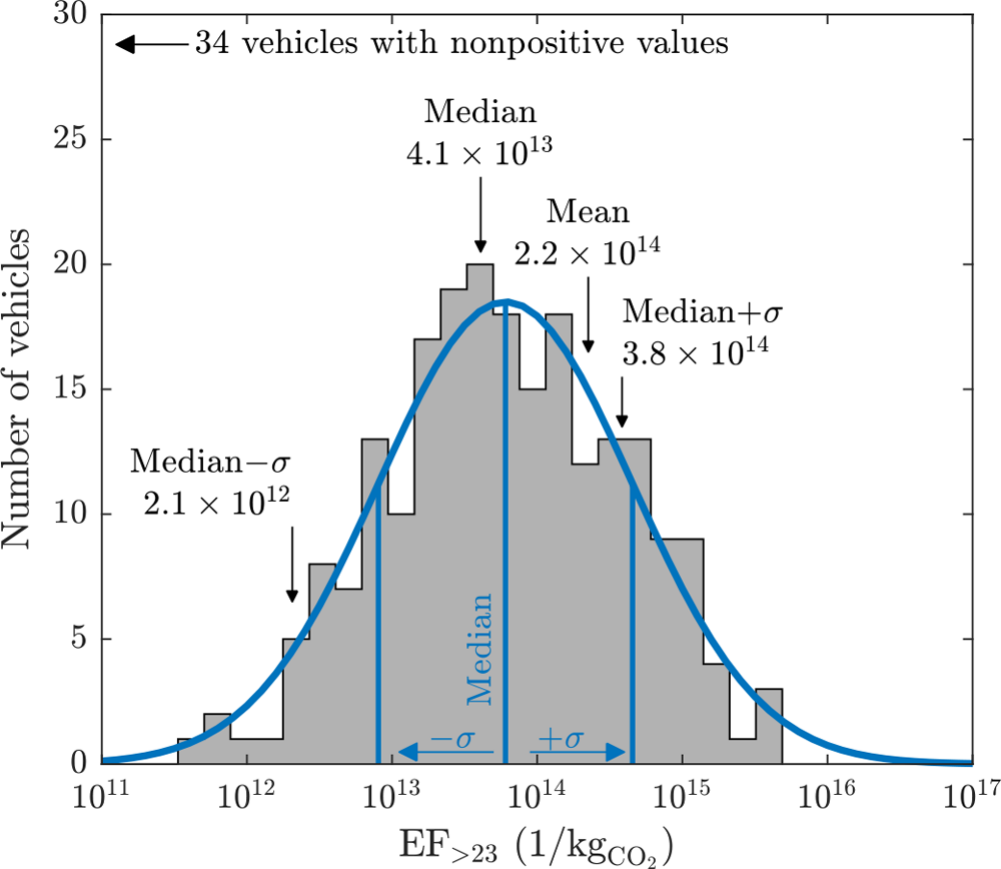
Distribution of emission factors of particles larger than 23 nm (EF_>23_) of all vehicles. Only positive EFs are shown. A log-normal distribution is fitted over the positive EF_>23_ distribution; the median and standard deviation (σ) lines are also shown. Arrows denote the locations of the parameters when also the 34 non-positive EFs are included.

EF_>23_ from all vehicles are also expressed as a distribution in Figure 4. It can be observed that they follow closely a log-normal distribution, i.e., a normal distribution curve can be fitted over the EF_>23_ histogram when the EF_>23_ axis is in log-scale. Distributions of a similar type are observed also with other particle size ranges utilized here and in other vehicle chase studies.^31,41^ The vehicles with non-positive EF_>23_ are not shown in the histogram because they cannot be properly presented in log-scale.

The mean and median of the EF_>23_ for the whole vehicle fleet are 2.2 × 10^14^/kg_CO_2__ and 4.1 × 10^13^/kg_CO_2__, respectively. The median ± σ values represent the standard deviations to the left and to the right from the median or, in other words, the 15.87th and 84.13th percentiles. The mean value is usable in estimating the total particle emissions of the whole fleet (driving under summer conditions on a highway or a regional road) affecting the outdoor particle concentrations, e.g., for regional- or global-scale aerosol models. The median value, instead, represents the emissions of a typical, or an average, vehicle.

### Emission Factors of Vehicles of Different Types and Emission Levels

**Figure 5:**
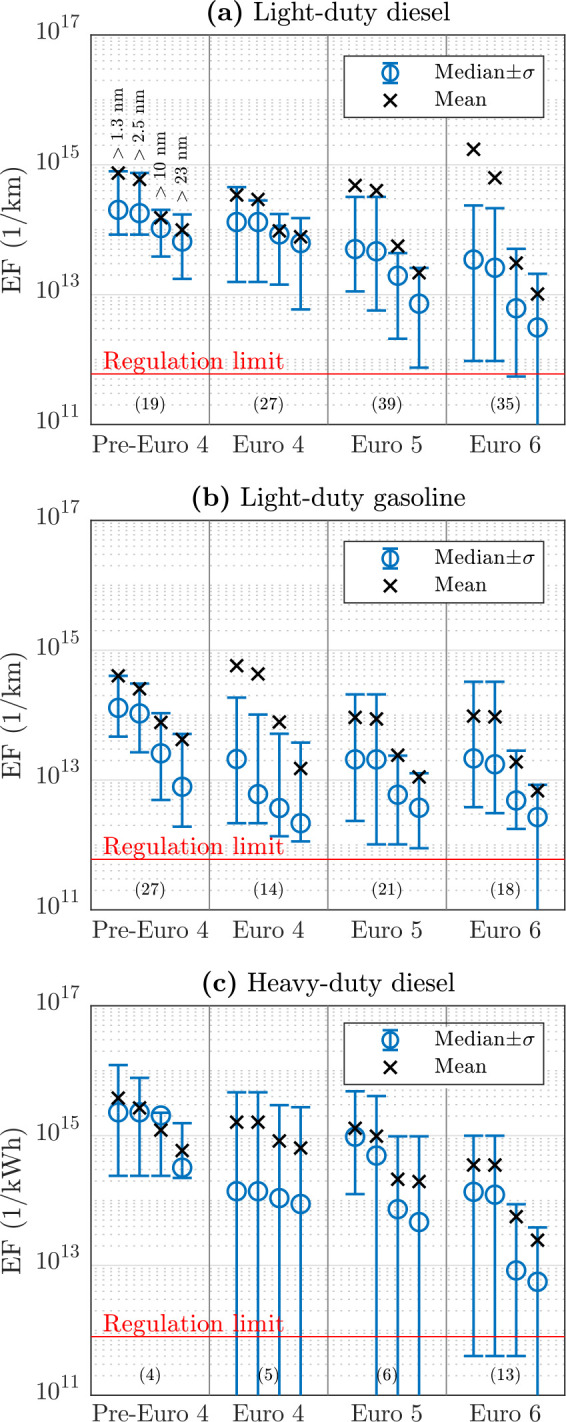
Particle number emission factors (EFs) of (a) light-duty diesel, (b) light-duty gasoline, and (c) heavy-duty diesel vehicles with different emission levels. The EF data are shown as the medians (with the standard deviations, σ) and means for the vehicle categories, of which sample sizes are marked in the parentheses. EFs are expressed for four different particle size ranges, shown in the first panel. The red lines represent the limits of particle number emissions in chassis dynamometer tests utilized with the most recent regulation levels.

The emission factors in the units of 1/kg_CO_2__ are converted to PN emitted by 1 km driving (LD vehicles) or by 1 kWh engine work (HD vehicles) to express them in similar units to the regulation limits (see the Supporting Information Text S2.4 for the details of the conversions). The statistics of the obtained EFs for the particle size ranges (>1.3, >2.5, >10, and >23 nm) are presented graphically in Figure 5 and numerically in Table S5, separately for different fuel and vehicle types and for different regulation levels. Table S5 presents the data also for the particle size bins (1.3–2.5, 2.5–10, and 10–23 nm). All negative EFs are forced to zero at this point because they cannot be properly presented in log-scale and because negative values cannot be used in calculating EFs for the size bins and in performing the diffusional losses corrections. Twenty-five vehicles are omitted here either because their regulation levels in the national database—mainly due to the vehicles being registered abroad—are missing (14 vehicles) or because they are vehicles with bifuel or hybrid technologies (11 vehicles). It can be observed that particle emissions have generally decreased with the advancement of the regulation levels, but most of the vehicles emit remarkably more PN than the PN limits given in Euro 5b or 6 levels. Even most of the Euro 6 vehicles exceed the limit although the limit applies to them (except non-GDI gasoline vehicles). However, the limit considers only non-volatile particles larger than 23 nm. Thus, the PN regulated vehicles exceeding the limit for any other particle size range than for >23 nm do not necessarily violate the regulation limits, and the emissions of >23 nm particles can also be partly due to semi-volatile—and thus unregulated—particles. However, many vehicles presumably violate their non-volatile particle limits in their realistic operation because >23 nm particles are mainly non-volatile soot particles.^47^ A study with the Finnish vehicle fleet^6^ shows that 65% of the number of vehicle-emitted >23 nm particles contain a non-volatile core; albeit the study was performed in a street canyon with lower vehicle speeds, it can be estimated that the non-volatile limits are exceeded by many vehicles also in highway driving because the median EF_>23_ values exceed the limits by almost an order of magnitude. One reason for the limit-exceeding particle emissions is that aged vehicles emit more particles than when they were new.

Decreasing PN emissions with advancing technologies, or regulation levels, can be seen most clearly for diesel vehicles, especially for >23 nm particles. Vehicles with the most recent regulation levels include many vehicles for which negative EF_>23_ values were obtained, which can be seen as zero median−σ values. This could be explained with the introduction of particle filters. For gasoline vehicles, no significant improvement in decreasing PN emissions have been obtained with advancing regulation levels. This is seen especially for the total PN (>1.3 nm). Additionally, there are some Euro 6 LD diesel vehicles emitting a significant number of particles larger than 1.3 or 2.5 nm, which can be seen as very high mean values. Those specific vehicles can even be the dominant emitters of very small, say sub-10 nm, particles of all LD vehicles, although they belong to the strictest regulation level.

Size distributions of the emitted particles can be inspected from the trends of the EFs with respect to the particle size in Figure 5. The mean of NCA fractions (PN emission of a vehicle within the size range 1.3–2.5 nm divided by the total PN emission of the vehicle) is 12% for the diesel vehicles and 18% for the gasoline vehicles. Instead, the median of the NCA fractions for all vehicles is 0%, denoting that NCA emissions have been detected only for less than half of the vehicles (see Table S5). However, because some high emitters are included in the sample of studied vehicles, the fraction of all NCA emissions to the total PN emission from all LD vehicles is as high as 42%. It should be kept in mind that determining NCA levels generally involves very high relative uncertainty (−43%/+104% in this study), mainly due to uncertainties in correcting diffusional losses for such a low particle size range (see the Supporting Information Text S1.2). It should also be noted that the NCA levels reported in earlier studies with sampling systems of the similar type to the one in this study—especially when diluters are used—may be underestimated with the factor of up to ∼2 when compared to the levels reported in this study, due to novel diffusional loss correction methods (see the Supporting Information Text S1.2) applied in this study but do typically lack in earlier studies. For >23 nm particles, the mean of the fractions are 36% for diesel vehicles and 24% for gasoline vehicles. Because most of the emitted particles fall in sizes below 23 nm, the contribution of delayed primary particles is apparently high. However, it should be noted that even the NCA emissions have been observed to contain 32% of non-volatile cores;^6^ therefore, all sub-23 nm particles are not necessarily delayed primary particles.

Eighty-two percent of the total number of >1.3 nm particles emitted by all LD vehicles were observed to originate from diesel vehicles, whereas LD diesel vehicles had the share of 60% in the chase experiments. For NCA, this contribution is 88%. Examining the LD diesel vehicle category more deeply, it can be observed that 84% of NCA is emitted by Euro 6 level vehicles, within this category, although their share in the chase experiments, within this category, was only 29%. For >23 nm particles instead, this contribution of Euro 6 level LD diesel vehicles to all LD diesel vehicles is only 6.9%, suggesting that the DPFs operate properly, on average. However, they seem to be not efficient in reducing emissions of very small particles, such as NCA. Euro 6 level gasoline vehicles, instead, have the contribution of only 0.7% to NCA emissions from all gasoline vehicles, whereas the share of Euro 6 level vehicles in the chase experiments was 23%, within this category.

In conclusion, most of the studied vehicles exceed the PN regulation limits by 1–2 orders of magnitude in real-world driving. For HD vehicles, even exceedings of 3 orders of magnitude are common. For LD and HD diesel vehicles, the PN emissions are lower with newer regulation levels, implying some success of developed PN emission controlling because the regulation limits began to apply not until the Euro 5 level.

### Comparing the Emission Factors with Other Studies

High PN emissions from vehicles in their real operation have been observed in other studies as well (see Table S6). Hietikko et al.^3^ observed that, in 2017, EF_>1.3_ was 130 × 10^13^/kg_CO_2__ in a highly trafficked urban street canyon in Helsinki, Finland, representing the mean emissions from all vehicles passing by the measurement station at a fixed location. Lintusaari et al.^6^ observed EF_>1.3_ of 110 × 10^13^/kg_CO_2__ and EF_>23_ of 5.4 × 10^13^/kg_CO_2__ in 2018 at the same location. For comparison, the means (excluding the omitted 25 vehicles) of EF_>1.3_, 380 × 10^13^/kg_CO_2__, and of EF_>23_, 25 × 10^13^/kg_CO_2__, were observed in this study. These are up to almost 5 times higher than the values observed in the street canyon studies. The studies are, however, not directly comparable because the street canyon studies represent emissions from urban driving whereas this study uses mostly the data from highway driving.

A comparison to EFs from highway driving can be done using the results of a curbside measurement by Ban-Weiss et al.,^33^ who reported the mean >3 nm particle EF of 150 × 10^13^/kg_CO_2__ for 224 HD vehicles driving in an uphill highway tunnel in California in 2006. In this study, the mean EF_>2.5_ of 180 × 10^13^/kg_CO_2__ for the HD vehicles was obtained. These EFs are comparable although the vehicle fleets between the US and Europe and between the years 2006 and 2020 differ substantially, with the older fleet (US) showing even lower emissions although it was driving uphill, i.e, with high engine loads.

A Slovenian chase measurement study in 2011 by Ježek et al.^31^ reports the mean >5.6 nm particle EF of 140 × 10^13^/kg_CO_2__ for diesel vehicles and of 62 × 10^13^/kg_CO_2__ for gasoline vehicles, which are comparable to the EFs obtained in this study because they lay between the mean EF_>2.5_ and EF_>10_ values.

A literature review by Giechaskiel et al.,^48^ combining EFs from several studies with gasoline vehicles (including also some Euro 6 level vehicles), reports EFs of all particles larger than typically 2.5–6 nm in the range of about 10^11^/km to 10^13^/km for non-GDI gasoline vehicles and in the range of about 10^12^/km to 10^14^/km for GDI vehicles, whereas this study shows that EF_>1.3_, EF_>2.5_, and EF_>10_ for gasoline vehicles are mainly in the range of about 10^12^/km to 10^14^/km.

### Implications to the Development of Particle Emission Regulations

In conclusion, as the results show, the mean NCA emissions of the newest LD diesel vehicles are dominated by few high-emitting vehicles. Thus, controlling the particle emissions during the vehicle life cycle is important in preventing the contribution of high-emitting single vehicles to the total emissions from the whole vehicle fleet. Even the latest additions to periodical technical inspections controlling the number concentration of >23 nm particles do not catch the high emissions of the smallest particles. Thus, also sub-10 nm particles should be recognized in developing future regulations to reduce the total PN concentrations in ambient air, although some challenges exist in the execution.

There is a fundamental question whether the regulations should focus on all particles or on non-volatile particles only. Because semi-volatile particle emissions depend greatly on the conditions outdoor, controlling only non-volatile particles is clearly easier to implement, which, however, results in real-world particle emissions being higher than the regulation limits. Correction factors used to correct particle losses in the sampling systems increase steeply with decreasing particle size below 10 nm, especially below 3 nm, when systems consisting of several stages are used (see Table S1). It is impossible to obtain a particle size-dependent correction factor with only one particle counter during a fast measurement but would be feasible with several particle counters in parallel, however, with increased system cost. One simplified approach would be to allow for different diffusional losses for different particle sizes; hence, the smallest particles would be subjected to smaller weight factors. This could still be somewhat efficient since the smallest particles in the exhaust plumes are often found in very high concentrations.

## Supporting Information Available

Discussions of data processing details and determining emission factors of particles using CO_2_ concentration data, tables of correction factors for different particle size ranges, comparison of the integration and slope methods for the example chase events, equations for different versions of emissions factors, particle number emission factors of vehicles with different emission levels, and particle number emission factors compared to other studies, and figures of time series and scatter plots from two example chase events and emission factors of particles larger than 23 nm using ordinary regression, (PDF)
