# Supplementary material for: High Particle Number Emissions Determined with Robust Regression Plume Analysis (RRPA) from Hundreds of Vehicle Chases
Source: Environ Sci Technol. 2023 Jun 7;57(24):8911–20. doi: 10.1021/acs.est.2c08198 (PMC10286313; doi:10.1021/acs.est.2c08198)
Supplement: Supplementary file 1 — es2c08198_si_001.pdf [file es2c08198_si_001.pdf]

Supporting Information: High Particle Number Emissions Determined with Robust Regression Plume Analysis (RRPA) from Hundreds of Vehicle Chases

Miska Olin,[*,†] Henri Oikarinen,[‡] Petteri Marjanen,[†] Santtu Mikkonen,[‡,¶] and Panu Karjalainen[†,§]

†*Aerosol Physics Laboratory, Tampere University, FI-33014 Tampere, Finland*

‡*Department of Applied Physics, University of Eastern Finland, FI-70211 Kuopio, Finland*

¶*Department of Environmental and Biological Sciences, University of Eastern Finland, FI-70211 Kuopio, Finland*

§*Institute for Advanced Study, Tampere University, FI-33014 Tampere, Finland*

E-mail: miska.olin@tuni.fi

This Supporting Information document contains:

23 pages, 2 texts, 6 tables, and 2 figures.

S1 Data Processing Details

S1.1 Particle Size-Independent Corrections

All measurement devices of which data are used in this study have time responses of ∼1 s and they also record the data in 1 s time resolution. Due to different sample residence times in the sampling lines for different devices and due to possible differences between the clocks determining the time stamps for the recorded data, the measurement data were first synchronized by using peaks in time series data from flame tests performed several times during the measurements. The flame tests were conducted by bringing liquefied petroleum gas flames near the tip of the inlet of the measurement van for short periods. Due to a small uncertainty in obtaining an accurate time synchronization between the devices, the measurement data were also averaged into 3 s time resolution before further analysis.

All measured particle concentrations were multiplied with the dilution ratio (DR) of the used bifurcated flow diluter set. This type of diluter directs the major part of the flow directly through a HEPA filter and only a small part past the filter. The proportion of the total flow rate to the flow rate bypassing the filter basically determines the DR of the diluter. The total DR of the diluter set consisting of two similar diluters in series for >50 nm particles was measured to be 98±4 by having a source of a test dioctyl sebacate aerosol with a known concentration upstream of the diluter set and by measuring the concentration downstream. The measured particle concentrations were also corrected for the maximum detection efficiencies of the CPCs. These were also obtained from the dioctyl sebacate aerosol measurement, where the particle size distribution was measured and was with enough large particles. The obtained maximum detection efficiencies are 72.84% for the PSM+CPC combination (> 1.3 nm), 100.4% for the CPC > 2.5 nm, 82.89% for the CPC > 10 nm, and 99.91% for the CPC > 23 nm. The detection efficiencies of the CPCs decrease with decreasing particle size close to their cut-off diameters, but they are handled here by assuming step functions where the detection efficiency is zero for particles smaller than the cut-off diameter

and the previously mentioned maximums for particles larger than the cut-off diameter.

S1.2 Particle Size-Dependent Corrections

Traditionally, experiments including ultrafine particles have required corrections to the measured particle concentrations due to diffusional losses of particles onto the inner walls of the sampling lines. These corrections involve particle size-dependent correction factors which the measured concentrations need to be multiplied with. Here we calculated these correction factors using the functions by Gormley and Kennedy[S1] (for straight tubes with laminar flows) and Brockmann[S2] (for straight tubes with turbulent flows). The sampling system for the CPCs consisted of 4.5 m of tubes in total. The first part was a fixed 2.5 m line installed in ATMo-Lab. The flow rate through this main line with the inner diameter of 9 mm was 30 slpm, denoting turbulent flow. The next part of the sampling system was 1 m line with the inner diameter of 6 mm. This line was used for the CPCs; the flow rate being 6.6 slpm, denoting laminar flow. The last part consisted of separate parallel 1 m lines with the inner diameters of 4 mm for each CPCs. All the last lines had laminar flow, the flow rates depending on the CPC (2.5 slpm for the PSM+CPC combination ($>$ 1.3 nm), 1.5 slpm for the CPC $>$ 2.5 nm, and 1 slpm for the rest). There were four sharp bends in these lines in total; two in the main line, one in the next line, and one in each of the last parallel lines. Because the front inlet was pointing downwards to prevent excessive dust passing the measurement system, the sampling was not isokinetic, but it could have not been achieved anyway because the driving speed was not a constant. By taking also aspiration, gravitational losses, and inertial losses in the bends into account, aspiration becomes notable with particles larger than 100 nm and inertial losses in the bends with particles larger than 1000 nm. For example, the aspiration efficiency of 100 nm particles, according to Hangal and Willeke[S3], is 99% with the driving speed of 60 km/h and 94% with the driving speed of 120 km/h. Nevertheless, because the major fraction of particle number in vehicles emissions is typically in particle sizes much smaller than 100 nm and this study focuses on particle

number only, these sampling system effects on larger particles were not corrected.

The obtained correction factors (omitting the effects on large particles) for the four particle size ranges of this study can be found in Table S1, from which it can be seen that the correction is significant especially for the smallest particle sizes. In addition to particle sizes, these corrections also depend, e.g., on the lengths and inner diameters of the tubes and the flow rates, of which determination involves some measurement uncertainties, but the largest uncertainty arises from the selection of the representative particle size within a specific particle size range. The typically selected geometric mean diameter of a size range may not truly represent the particle size with which the calculations should be done due to non-linearity in the correction functions. The representative particle size of a size range depends also on the shape of the particle size distribution within the size range, which cannot be easily determined. We calculated also the ranges of variation for the correction factors by assuming that the variation is caused only by the selection of the representative particle size.

Table S1: Correction factors for different particle size ranges. The first values in the cells represent the factors which are applied to the measured data and they are calculated using the geometric mean diameter of a size range, i.e., the 50th percentile of a size range in log-scale (for example, 1.8 nm for the size range of 1.3–2.5 nm). The values in parentheses represent ranges of variation for the factors, calculated by assuming that the representative particle size within a size range can vary between the 25th and the 75th percentile of a size range in log-scale (for example, 1.53 and 2.12 nm for the size range of 1.3–2.5 nm). For the size range of >23 nm particles, the factors are calculated by selecting 50 nm for the mean diameter and 23 nm and 100 nm for calculating the ranges of variation.

Corrections	1.3–2.5 nm	2.5–10 nm	10–23 nm	>23 nm
**Traditional**				
Straight tubes	7.772 (5.14–13.2)	1.793 (1.44–2.53)	1.163 (1.12–1.22)	1.034 (1.01–1.09)
**Novel**				
Bends in tubes	1.132 (1.11–1.16)	1.030 (1.02–1.05)	1.003 (1.00–1.01)	1.001 (1.00–1.01)
Diluters	1.863 (1.65–2.18)	1.172 (1.11–1.28)	1.038 (1.03–1.05)	1.008 (1.00–1.02)
Subtotal	2.108 (1.82–2.54)	1.207 (1.12–1.35)	1.041 (1.03–1.06)	1.009 (1.00–1.02)
**Total**[1]	16.39 (9.35–33.5)	2.163 (1.62–3.41)	1.211 (1.16–1.29)	1.043 (1.02–1.12)
**Total**[2]	16.39 (−43%/+104%)	2.163 (−25%/+58%)	1.211 (−4.5%/+6.3%)	1.043 (−2.3%/+7.1%)

[1] Ranges of variation as numbers. [2] Ranges of variation as percentages of the factors, i.e, relative to the factors.

Novel methods to calculate the corrections were also applied in this study. These include correcting elevated diffusional losses of particles in bent parts of the sampling lines and correcting the DR of the bifurcated flow diluters for very small particles (due to increased diffusional losses). Bends in tubes also increase losses of larger particles due to particle impaction on the inner walls but this effect was omitted in this study, since it is negligible for the ultrafine particle size range of interest in this study. The correction factors for the diffusional losses in the bends (four sharp bends) in the sampling lines were calculated using the functions by Olin and Dal Maso [S4]. It can be seen from Table S1 that this effect is notable only for the lowest particle size range (1.3–2.5 nm).

The DR of the used bifurcated flow diluter set was determined only for particles larger than 50 nm, but, in reality, DR is higher for the smaller particles due to increased diffusional losses in diluters. This elevated DR can be corrected by multiplying the results with another diffusional losses-based correction factor. Here we used a method used by Collins [S5], in which the diffusional losses inside a diluter are experimentally determined for different particle sizes and the obtained penetration vs. particle diameter curve is used to find L/Q, the ratio of the bypassing tube length (L) and the bypassing flow rate (Q), producing this curve with the function by Gormley and Kennedy [S1]. The diluter set used in this study was not tested for smaller particles, but here we use the losses data determined with silver particles for another similar diluter. The smallest particles used in the test were about 3 nm in diameter, because handling even smaller particles accurately becomes very difficult. The fitted L/Q value obtained from the test (with one diluter with the total flow rate of 2.5 slpm and DR of 12, denoting Q of 0.21 slpm) is $0.46\,\mathrm{m\,slpm^{-1}}$, resulting in $L = 96\,\mathrm{mm}$, which is much longer than the physical length of the bypassing tube in the diluter. This suggests that the function by Gormley and Kennedy [S1] do not suit very well for this kind of diffusional losses calculation (because it is derived only for straight tubes with some additional assumptions), but is, however, the best option currently available. On the other hand, the curve of the function by Gormley and Kennedy [S1] fits surprisingly well onto the experimental data in the

study by Collins[S5], in which the lowest particle diameter was 9 nm. However, predicting the losses for the smaller particles requires extrapolation, of which correctness cannot be easily verified. Additionally, the obtained L value of a diluter may depend also on the flow rate (during the chase experiment, it was 6.6 slpm, instead of 2.5 slpm during the diffusional losses test). Table S1 showing the obtained correction factors implies that the effect of the diffusional losses of particles in the diluter set is notable for the lowest particle size range but the correction should not be neglected even for the next size range (2.5–10 nm).

It can be seen from the total correction factors in Table S1 that the lowest particle size range required remarkable corrections, the total correction factor being 16.39. The uncertainty of this correction factor is also high, −43%/+104%, meaning that the concentrations of 1.3–2.5 nm particles reported in this study may be over- or under-estimated with the factor of about 2 (the reported concentrations can be decreased 43% or increased 104%, in maximum). The uncertainty is still relatively very high also for the next particle size range, 2.5–10 nm, (−25%/+58%) but the correction factor itself is already much lower (2.163).

It should be noted that because these novel correction methods (diffusional losses due to bends in tubes and diluters) have not been applied in most of the studies in literature, the concentrations reported in this study may not be directly comparable with previous studies reporting only the lower limits of the concentrations. The subtotal correction factors in Table S1, denoting the effect of the novel correction methods, can be used to make the results of this study comparable. The subtotal correction factor for, e.g., 1.3–2.5 nm particles is 2.108, denoting that the concentrations of 1.3–2.5 nm particles reported here should be divided by 2.108 in order to compare them with studies where these novel corrections have not been applied but the measurement systems yet contained (a similar number of) bends in tubes and (similar bifurcated flow) diluters. It should, however, be noted that these corrections are system-specific and may be totally neglected with some measurements systems (using only straight tubes and no diluters), but the magnitude of them (∼2.1 for the 1.3–2.5 nm particles, ∼1.2 for the 2.5–10 nm particles, and negligible for particles larger than ∼10 nm)

should be kept in mind.

S2 Determining Emission Factors of Particles Using CO_2 Concentration Data

S2.1 Instantaneous Emission Factors

First, the method for deriving instantaneous emission factors (EFs) is presented. They hold only for a given time range, on the order of a few seconds, including time spent by aspiration of the background air by the engine, the combustion process, and transferring of the exhaust within the exhaust system and in the background air—after releasing from the tailpipe—to the location where the exhaust sample is measured.

Let a particle number concentration (in $1/\mathrm{cm}^3$ in NTP conditions) be N_{raw} in the raw exhaust, N_{bg} in the background air, and N_{m} measured at any location. The measured concentration is thus

$$N_{\mathrm{m}} = fN_{\mathrm{raw}} + (1-f)N_{\mathrm{bg}} \tag{S1}$$

where f denotes the volume fraction of the raw exhaust for a given measurement location (see, e.g., Herndon et al.[S6]). Eq S1 can also be expressed as

$$f = \frac{N_{\mathrm{m}} - N_{\mathrm{bg}}}{N_{\mathrm{raw}} - N_{\mathrm{bg}}}. \tag{S2}$$

By using the assumption that particles dilute within a turbulent exhaust plume with rates equal to CO_2,[S7] eq S2 leads to

$$\frac{N_{\mathrm{m}} - N_{\mathrm{bg}}}{N_{\mathrm{raw}} - N_{\mathrm{bg}}} = \frac{[\mathrm{CO_2}]_{\mathrm{m}} - [\mathrm{CO_2}]_{\mathrm{bg}}}{[\mathrm{CO_2}]_{\mathrm{raw}} - [\mathrm{CO_2}]_{\mathrm{bg}}} \tag{S3}$$

where $[\mathrm{CO_2}]_{\mathrm{raw}}$, $[\mathrm{CO_2}]_{\mathrm{bg}}$, and $[\mathrm{CO_2}]_{\mathrm{m}}$ denote the CO_2 concentrations (in ppm) in the raw exhaust, in the background air, and in the measured sample, respectively.

Emission factor (EF) in the units of number of emitted particles per 1 kg of emitted CO_2 is defined as

$$\mathrm{EF} = \frac{E_\mathrm{N}}{E_{\mathrm{CO_2}}} = \frac{\dot{V} \times N_\mathrm{raw}}{\dot{V} \times [\mathrm{CO_2}]_\mathrm{raw} \times 10^{-6}\,\mathrm{ppm}^{-1} \times \rho_{\mathrm{CO_2}}} \tag{S4}$$

where E_N is the emission rate of particles (in 1/s), $E_{\mathrm{CO_2}}$ is the emission rate of CO_2 (in kg/s), $\dot{V}$ is the volumetric flow rate of exhaust (in cm^3/s in NTP conditions), $10^{-6}\,\mathrm{ppm}^{-1}$ comes from the conversion of ppm to unity, and $\rho_{\mathrm{CO_2}}$ is the density of CO_2 ($= 1.83 \times 10^{-6}\,\mathrm{kg/cm^3}$ in NTP conditions). After cancelling $\dot{V}$ out and substituting the value for $\rho_{\mathrm{CO_2}}$, eq S4 becomes

$$\mathrm{EF} = \frac{N_\mathrm{raw}}{[\mathrm{CO_2}]_\mathrm{raw}} \times 5.47\ \times 10^{11}\,\mathrm{cm^3\,ppm/kg_{CO_2}}. \tag{S5}$$

Many studies (e.g., Herndon et al.[S6], Zavala et al.[S8]) utilize the approximations,

$$[\mathrm{CO_2}]_\mathrm{raw} - [\mathrm{CO_2}]_\mathrm{bg} \approx [\mathrm{CO_2}]_\mathrm{raw} \tag{S6}$$

$$N_\mathrm{raw} - N_\mathrm{bg} \approx N_\mathrm{raw}, \tag{S7}$$

to simplify eq S3 to

$$\frac{N_\mathrm{m} - N_\mathrm{bg}}{[\mathrm{CO_2}]_\mathrm{m} - [\mathrm{CO_2}]_\mathrm{bg}} = \frac{N_\mathrm{raw}}{[\mathrm{CO_2}]_\mathrm{raw}}, \tag{S8}$$

which is the first part of the function for EF. Hence, EF can be obtained by dividing the excess particle number concentration ($N_\mathrm{m} - N_\mathrm{bg}$) with the excess CO_2 concentration ($[\mathrm{CO_2}]_\mathrm{m} - [\mathrm{CO_2}]_\mathrm{bg}$). The first approximation (eq S6) is generally valid since $[\mathrm{CO_2}]_\mathrm{raw}$ is on the order of 100,000 ppm and $[\mathrm{CO_2}]_\mathrm{bg}$ on the order of 400 ppm. The latter approximation (eq S7) is also typically valid with many pollutants, but not always with particle emissions, as is the case here, since particle concentrations in raw exhaust can be close to the background concentrations or even close to zero, e.g., for vehicles equipped with particle filters. Therefore, we utilize here a slightly different approach in deriving EF. Firstly, since the background air aspirated by the engine already contains CO_2 (with the concentration of $[\mathrm{CO_2}]_\mathrm{bg}$), its concentration in exhaust, $[\mathrm{CO_2}]_\mathrm{raw}$, has not entirely originated from combustion, but

$[CO_2]_{raw} - [CO_2]_{bg}$ has instead. Hence, $[CO_2]_{raw}$ should be replaced with $[CO_2]_{raw} - [CO_2]_{bg}$ in eq S5. Secondly, because the flow rates of exhaust and intake air are almost equal, the net contribution of a vehicle to the atmospheric particle number concentration is $N_{raw} - N_{bg}$ instead of N_{raw}. In other words, only the increase of the pollutant loading in a fixed air parcel aspirated by the engine and released from the tailpipe is consider emission. Now eq S5 becomes

$$\mathrm{EF} = \frac{N_{raw} - N_{bg}}{[CO_2]_{raw} - [CO_2]_{bg}} \times 5.47 \times 10^{11}\,\mathrm{cm^3\,ppm/kg_{CO_2}}. \tag{S9}$$

Eq S3 can be arranged to

$$\frac{N_m - N_{bg}}{[CO_2]_m - [CO_2]_{bg}} = \frac{N_{raw} - N_{bg}}{[CO_2]_{raw} - [CO_2]_{bg}}, \tag{S10}$$

which is the first part of eq S9. Eventually, both of these two approaches lead to the same function for EF,

$$\mathrm{EF} = \frac{N_m - N_{bg}}{[CO_2]_m - [CO_2]_{bg}} \times 5.47 \times 10^{11}\,\mathrm{cm^3\,ppm/kg_{CO_2}}. \tag{S11}$$

Because N_{raw} can be lower than N_{bg}, due to combusting particles existing in the background air aspirated by the engine and due to filtering particles formed during the combustion process, EF can be negative as well. By assuming a vehicle with zero particles in its exhaust is driving in the background particle concentration of $10^6\,\mathrm{cm^{-3}}$ (e.g., driving in an exhaust plume of a highly-emitting vehicle), we obtain the value for the most negative EF possible, which is on the order of $-10^{13}/\mathrm{kg_{CO_2}}$.

S2.2 Averaged Emissions Factors

By simply averaging EFs from a longer time range, such as from a single chase event, does not output a real EF-average because the exhaust flow rate, $\dot{V}$, can vary. Therefore, averaging by using $\dot{V}$ values as weights would be needed (see Wihersaari et al.[S9]), but is typically

unavailable in the case of chasing a large number of vehicles on public roads.

In many studies involving exhaust plume measurements (e.g., Canagaratna et al.[S10], Ježek et al.[S11]), instantaneous EFs are averaged over the time spent in the plume by dividing the time integral of the excess pollutant concentration by the time integral of the excess CO_2 concentration, as originally proposed by Hansen and Rosen[S12],

$$\frac{\int (N_\mathrm{m}(t) - N_\mathrm{bg})\,\mathrm{d}t}{\int ([\mathrm{CO_2}]_\mathrm{m}(t) - [\mathrm{CO_2}]_\mathrm{bg})\,\mathrm{d}t}, \tag{S12}$$

where particle number concentrations are used here for consistency, instead of black carbon concentrations used in the original publication. We call this *the integration method* in this article. This method, however, outputs real EF-averages only if at least one of these two conditions are met: (1) EF is constant or (2) both f and $\dot{V}$ are constants, within the given time range. Because at least dilution (i.e., f) is not constant in chase measurements due to natural fluctuations in a turbulent exhaust plume and due to varying sampling position (the measuring vehicle is not constantly in the center line of the plume with a constant distance), using the integration method basically requires an assumption of constant EFs. Nevertheless, the method outputs quite acceptable EF-averages from long enough chasing events from nearly constant driving behavior (thus, nearly constant $\dot{V}$), where fluctuations of f can be assumed to be distributed around a constant value.

The integration method (eq S12) explicitly needs the background values, N_bg and $[\mathrm{CO_2}]_\mathrm{bg}$, which are also assumed constants and can have substantial effects on the calculated EFs, especially when EFs are low or dilution is strong. Because the background values cannot practically be measured simultaneously with measuring the exhaust sample in chase experiments, they are typically determined before and/or after the chase events with no other nearby traffic. This is always not possible, and uncertainties arise when the background concentrations are observed to differ between the data before and after the chase event or when determining them is not successful due to other nearby traffic or due to drifting background

measurement data. Differing and drifting data can be caused, e.g., when the surrounding area changes during the measurement from a forest area to a field area or vice versa. Using eq S12 and the assumption of constant background concentrations, EFs can be calculated with the equation

$$\mathrm{EF} = \frac{\langle N_\mathrm{m} \rangle - N_\mathrm{bg}}{\langle [\mathrm{CO_2}]_\mathrm{m} \rangle - [\mathrm{CO_2}]_\mathrm{bg}} \times 5.47 \times 10^{11}\,\mathrm{cm^3\,ppm/kg_{CO_2}}, \tag{S13}$$

where $\langle N_\mathrm{m} \rangle$ and $\langle [\mathrm{CO_2}]_\mathrm{m} \rangle$ are the time-averages of $N_\mathrm{m}(t)$ and $[\mathrm{CO_2}]_\mathrm{m}(t)$, respectively.

Another method to determine average-EFs from chase events without information on $\dot{V}$ is *the slope method.* The assumption of a constant EF within a chase event is needed also in the slope method. In addition to a particle number concentration measured at a specific time moment, N_m, let a particle number concentration measured at another time moment be $N_{\mathrm{m}'} = N_\mathrm{m} + \Delta N$, and similarly for the CO_2 concentrations, $[\mathrm{CO_2}]_{\mathrm{m}'} = [\mathrm{CO_2}]_\mathrm{m} + \Delta[\mathrm{CO_2}]$. Using the assumption of a constant EF, EFs are equal for the both time moments. Based on eq S11, this leads to an equation (also the background concentrations are assumed constants),

$$\frac{N_\mathrm{m} - N_\mathrm{bg}}{[\mathrm{CO_2}]_\mathrm{m} - [\mathrm{CO_2}]_\mathrm{bg}} = \frac{N_{\mathrm{m}'} - N_\mathrm{bg}}{[\mathrm{CO_2}]_{\mathrm{m}'} - [\mathrm{CO_2}]_\mathrm{bg}}, \tag{S14}$$

which, by substituting $N_{\mathrm{m}'}$ and $[\mathrm{CO_2}]_{\mathrm{m}'}$, becomes

$$\frac{N_\mathrm{m} - N_\mathrm{bg}}{[\mathrm{CO_2}]_\mathrm{m} - [\mathrm{CO_2}]_\mathrm{bg}} = \frac{N_\mathrm{m} + \Delta N - N_\mathrm{bg}}{[\mathrm{CO_2}]_\mathrm{m} + \Delta[\mathrm{CO_2}] - [\mathrm{CO_2}]_\mathrm{bg}}. \tag{S15}$$

By arranging the terms in eq S15, we obtain

$$\Delta[\mathrm{CO_2}] \times (N_\mathrm{m} - N_\mathrm{bg}) = \Delta N \times ([\mathrm{CO_2}]_\mathrm{m} - [\mathrm{CO_2}]_\mathrm{bg}), \tag{S16}$$

and further,

$$\frac{N_\mathrm{m} - N_\mathrm{bg}}{[\mathrm{CO_2}]_\mathrm{m} - [\mathrm{CO_2}]_\mathrm{bg}} = \frac{\Delta N}{\Delta[\mathrm{CO_2}]} = \frac{\partial N}{\partial[\mathrm{CO_2}]}. \tag{S17}$$

By combining eqs S11 and S17, we finally obtain

$$\mathrm{EF} = \frac{\partial N}{\partial[\mathrm{CO_2}]} \times 5.47 \times 10^{11}\,\mathrm{cm^3\,ppm/kg_{CO_2}}, \tag{S18}$$

which denotes that the EFs can be calculated with the slope in a N vs. $[CO_2]$ plot even without explicit information on the background concentrations of particle number and CO_2. When the both assumptions (a constant EF and constant background concentrations) are adequate for a chase event, the data in the N vs. $[CO_2]$ plot are, in theory, scattered so that the data points form a line. Otherwise (a varying EF and/or varying background concentrations), the slope of the linear fit on the data points represent one kind of an average EF for the chase event.

In this study, signals caused by other traffic in the vicinity of the chasing and the measured vehicle are neglected from the EF analysis using a robust regression method, in addition to the ordinary one. We selected the Matlab's default weight function, bisquare weighting, for robust regression. It uses an iteratively reweighted least squares algorithm, which sets the weights for all data points used in linear regression. It results in smaller weights for data points which are far from a fitted line, i.e., for outliers.

S2.3 Example Chase Events Analyzed with the Integration and Slope Methods

Figure S1 presents time series and scatter plots for number concentration of particles larger than 23 nm ($N_{>23}$) and for $[CO_2]$ measured in the vicinity of two example chase events. Example 1 (Fig. S1a,b) represents a case where the exhaust plume has been captured well (a clear increase in $[CO_2]$ during the chase) and where the measured vehicle has a high $EF_{>23}$ (a clear and extensive increase in $N_{>23}$). Example 2 (Fig. S1c,d) represents a case with a not very well captured plume and with lower $EF_{>23}$. It also demonstrates how the uncertainty in determining representative N_{bg} affects the calculated EF in a case where the

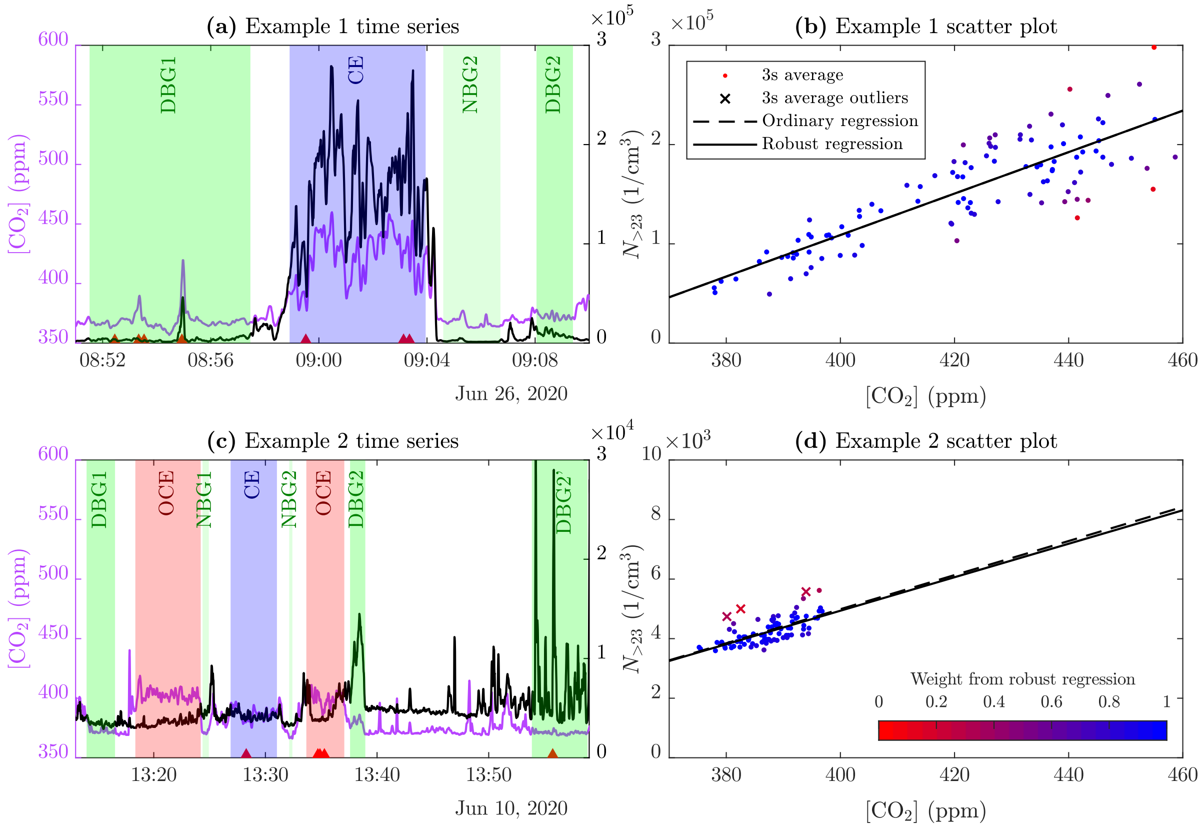

Figure S1: Time series and scatter plots from two example chase events. CE: chase event, DBG1/2: dedicated background measurement before/after the case event, NBG1/2: nearest background measurement right before/after the chase event, OCE: other chase event. Red pointers at the bottom of the time series plots denote moments with other vehicles passed/passing by. Note different scales of the $N_{>23}$ axes between the examples 1 and 2.

plume concentration, N_m, is very near the background level.

Time series data are used in determining EFs using the integration method. The variables in eq S13 are calculated as follows. $\langle N_m \rangle$ and $\langle [CO_2]_m \rangle$ are calculated as the means of the corresponding data within the blue-shaded area (chase event, CE), of which boundaries are determined via the researchers' decisions during the chase measurement (when the measurement van was positioned directly behind the studied vehicle with as constant chasing distance as possible and when no excessive changes in the driving speed occurred). Their uncertainties are determined here with the deviations in the means with the both boundaries altered ±2 s, representing the uncertainties of the decisions for the start and end times of the chase event and the possible uncertainty in the time synchronization between the both

measurement devices. Determining the background concentrations, N_{bg} and $[CO_2]_{\text{bg}}$, needs a careful assessment for choosing a representative time range that best covers data not affected by other traffic. Dedicated background measurements (DBG), in which the measuring vehicle was driven as far from other traffic as possible but still on the same road as the actual chase events, were performed between some of the chase events. Because these DBG measurements are not always performed right before or after the chase event, due to maximizing the coverage of different vehicle chases or due to traffic conditions then, background measurements nearest to the chase event (NBG) are also considered with these examples. These NBG measurements were, however, not performed intentionally but are, instead, more like measurements between chase events; thus, they may include data from other traffic. In example 1, there was this kind of a background measurement right after the chase event (NBG2), which was performed just before the dedicated background measurement after the chase event (DBG2). In example 2, there were only very short background-like measurements right before and after the chase event (NBG1 and NBG2), which are surrounded by data from other chase events (OCE), i.e., from other vehicles. The dedicated background measurements (DGB1 and DBG2) are right before and after these OCE measurements. Because the DBG2 measurement shows unexpectedly high particle concentration in example 2, also another dedicated background measurement (DBG2') is considered here. The background concentrations, N_{bg} and $[CO_2]_{\text{bg}}$, are calculated as the medians from the chosen background measurement time ranges. Their uncertainties are calculated as the corresponding standard deviations.

Determining EFs with the slope method (eq S18) requires only the slope, $\partial N / \partial [CO_2]$, which can be obtained from the linear fit (ordinary or robust regression) over the scatter plots (Fig. S1b,d). Standard errors of the slopes are considered their uncertainties. The data points in the scatter plots are averaged to 3 s for accounting possible uncertainties in the time synchronization between the devices.

The parameters and the results of the integration and the slope method for the example

chase events are presented in Table S2. The uncertainties in the EFs are calculated with the error propagation law from the uncertainties in the parameters. It can be seen that, in example 1, the both methods output EFs of similar magnitudes and that the chosen background time range does not greatly affect the results from the integration method. In example 2, the output EFs are very different between the methods, and the chosen background time range affects the result greatly. The uncertainties in the EFs output by the integration method are also relatively very high, mainly due to high relative uncertainties in N_{bg}. In conclusion, the slope method is superior to the integration method in example 2, where the integration method fails to output the EF with high certainty. The EFs output by the integration method in example 1 have higher certainty but they slightly depend on the chosen background measurement time range.

Table S2: Comparison of the integration and slope methods for the example chase events presented in Fig. S1. DBG denotes the background concentrations are calculated from the time range of dedicated background measurements before/after the chase event (DBG1/2). DBG' denotes that the data from DBG2' are used instead of DGB2 in the example 2. NBG denotes the background concentrations are calculated from the time range of the nearest background measurements right before/after the chase event (NBG1/2). All particle number concentrations refer to particles larger than 23 nm.

			Example 1	Example 2
**Integration method**				
$\langle N_{\mathrm{m}} \rangle$		$10^3/\mathrm{cm}^3$	155.56 ± 0.27	4.2537 ± 0.0029
$\langle [\mathrm{CO_2}]_{\mathrm{m}} \rangle$		ppm	422.000 ± 0.083	387.046 ± 0.026
N_{bg}	DBG	$10^3/\mathrm{cm}^3$	3.6 ± 6.1	3.6 ± 3.6
N_{bg}	DBG'	$10^3/\mathrm{cm}^3$	-	4.2 ± 4.8
N_{bg}	NBG	$10^3/\mathrm{cm}^3$	1.5 ± 1.1	4.2 ± 0.8
$[\mathrm{CO_2}]_{\mathrm{bg}}$	DBG	ppm	368.2 ± 7.9	373.8 ± 4.6
$[\mathrm{CO_2}]_{\mathrm{bg}}$	DBG'	ppm	-	371.2 ± 2.4
$[\mathrm{CO_2}]_{\mathrm{bg}}$	NBG	ppm	365.5 ± 4.2	370.1 ± 2.6
EF	DBG	$10^{13}/\mathrm{kg_{CO_2}}$	155 ± 24	2.52 ± 15
EF	DBG'	$10^{13}/\mathrm{kg_{CO_2}}$	-	0.26 ± 17
EF	NBG	$10^{13}/\mathrm{kg_{CO_2}}$	149 ± 11	0.11 ± 2.4
**Slope method**				
$\partial N/\partial [\mathrm{CO_2}]$	Ordinary	$\mathrm{cm}^{-3}/\mathrm{ppm}$	2090 ± 140	57.2 ± 7.0
$\partial N/\partial [\mathrm{CO_2}]$	Robust	$\mathrm{cm}^{-3}/\mathrm{ppm}$	2090 ± 140	56.2 ± 6.7
EF	Ordinary	$10^{13}/\mathrm{kg_{CO_2}}$	114.3 ± 7.2	3.13 ± 0.39
EF	Robust	$10^{13}/\mathrm{kg_{CO_2}}$	114.3 ± 7.4	3.08 ± 0.37

Overall, as the slope method does not explicitly need information on the background concentrations, it does not suffer from EFs depending on the chosen background measurement time range. It is also a very fast method because there is no need to examine the background measurement time ranges and because the robust regression method presumably neglects signals caused by other nearby vehicles. The slope method also seems to work well even when the EF is so low that the increase in the particle number concentration caused by the studied vehicle is very low (example 2). This capability of the slope method arises from the theories behind the methods: unlike in the integration method where the EF is calculated from the excess pollutant and [CO_2] concentrations with respect to the background concentrations (eq S13), the calculation of the EF in the slope method is based on the excess pollutant and [CO_2] concentrations with respect to their concentrations at "another time moments" (eqs S14–S17). Whereas the background concentrations for the integration method can only be measured at a different time from the chase event, the data from "the another time moments" for the slope method are measured continuously during the chase event.

The linear fits from the slope method should, in theory, point the background concentration of particle number or CO_2 if the background concentration of the other one is known. E.g., in example 1, the robust fitting line has a point with 350 ppm of [CO_2] and $4 \times 10^3/\text{cm}^3$ of $N_{>23}$. Those values could represent their background values. A possible explanation for higher EF output by the integration method is the median [CO_2] during the background measurements showing a too high value due to other nearby traffic. Nearly the mentioned concentration of 350 ppm is seen at about 08:54, which could represent a more realistic background concentration. Using the value of 350 ppm as $[CO_2]_{bg}$ in the integration method, gives the EF of $117 \times 10^{13}/\text{kg}_{CO_2}$, which is very near the EF output by the slope method ($114.3 \times 10^{13}/\text{kg}_{CO_2}$).

Table S3 presents similar comparison for the example chase events presented in Fig. 2. The integration method and the slope method with ordinary regression should give similar results. In many cases the uncertainties are so high that this expectation holds within the

Table S3: Comparison of the integration and slope methods for the example chase events presented in Fig. 2. All values refer to particle number emission factors of particles larger than 23 nm in unit $10^{13}/\mathrm{kg}_{\mathrm{CO_2}}$. The integration method is conducted using the dedicated background measurements. The cases where the integration and the slope method with ordinary regression agree are marked as bold values.

Fig. 2 subplot	Integration method	Slope method (ordinary)	Slope method (robust)
a	$\mathbf{92 \pm 13}$	$\mathbf{102 \pm 9}$	128 ± 6
b	30 ± 3	100 ± 30	5 ± 9
c	$\mathbf{3.2 \pm 0.7}$	$\mathbf{3 \pm 3}$	0.9 ± 0.8
d	250 ± 30	420 ± 110	10 ± 20
e	$\mathbf{150 \pm 30}$	$\mathbf{114 \pm 8}$	114 ± 8
f	$\mathbf{30 \pm 50}$	$\mathbf{-3 \pm 2}$	-0.6 ± 1.1
g	$\mathbf{-30 \pm 50}$	$\mathbf{2 \pm 3}$	4.3 ± 1.2
h	21 ± 11	-3 ± 11	1 ± 11

confidentiality limits (marked as bold). However, the issue of the selection of a representative background measurement causes that the results from the integration method can be deviated from the truth, causing weak comparability between the methods. Similarly to the slope method with ordinary regression suffering from outlying data points caused by external disturbances, the integration method can suffer from that too. The cases without the agreement between the integration method and the slope method with ordinary regression (subplots b, c, and h) are somewhat exceptional cases, where the outlying data points have very strong effect (subplots b and d) or where the exhaust plume was not captured very well (subplot h), as is discussed with Fig. 2. The slope method with robust regression, instead, omits these data points and thus leads to the lowest uncertainties. The agreement between the integration method and the slope method with robust regression cannot be simply evaluated because removing the external disturbances from the integration method is difficult.

S2.4 Converting Emission Factors from the Unit of $1/kg_{CO_2}$ to the Unit of $1/kg_{fuel}$, 1/km, or 1/kWh

Emission factors output by the slope method (eq S18) are in the unit of PN emitted per 1 kg of CO_2 emitted ($1/kg_{CO_2}$). They can be converted to PN emitted per 1 kg of fuel combusted ($1/kg_{fuel}$) by multiplying them with $3.16\,kg_{CO_2}/kg_{fuel}$ (for diesel vehicles) or $3.04\,kg_{CO_2}/kg_{fuel}$ (for gasoline vehicles). These coefficients are obtained from stoichiometric combustion calculations by assuming that all carbon atoms in the fuel produce CO_2 molecules, i.e., that the combustion process is perfect. Diesel is assumed to have the average chemical composition of $C_{12}H_{23}$. Gasoline is assumed to be a mixture which consists of 90 % of the average chemical compound C_8H_{15} and 10 % of ethanol (C_2H_6O), representing the most commonly used gasoline grade in Finland, 95E10. It should be noted that earlier studies typically utilize the same conversion coefficient (close to the one utilized here for diesel vehicles) for both diesel and gasoline vehicles because the coefficients have historically been almost equal. This is, however, not true anymore due to higher ethanol content in present gasoline grades. Thus, we utilize here separate conversion coefficients for diesel and gasoline vehicles and use the unit of $1/kg_{CO_2}$ when comparing the results with other studies.

EFs in the unit of $1/kg_{fuel}$ can be further converted to the unit of particle number emitted per 1 km driving (1/km) for light-duty (LD) vehicles if the fuel consumption is known. Here we use the median values of fuel consumptions from combined driving of all measured vehicles for which the values can be obtained from the national Traficom's database using their register numbers (6.1 l/100 km for LD diesel vehicles and 6.5 l/100 km for LD gasoline vehicles). The densities of the fuels are assumed to be $820\,kg/m^3$ for diesel and $748\,kg/m^3$ for gasoline,[S13] representing average road-use fuels used in Finland. For heavy-duty (HD) vehicles, EFs in the unit of $1/kg_{fuel}$ are converted to the unit of particle number emitted per 1 kWh of energy produced (1/kWh) by dividing the EFs by the heat value of diesel ($12\,kWh/kg_{fuel}$[S14]) and by the average of typical brake thermal efficiencies of HD vehicles, 43%–46%,[S15] (44.5%). The equations for all the versions of EFs are assembled in Table S4.

Table S4: Equations for different versions of emissions factors, i.e., with different units.

EF version	LD diesel	LD gasoline	HD diesel
$\mathrm{EF}[1/\mathrm{kg}_{\mathrm{CO_2}}] =$	$\frac{\partial N}{\partial[\mathrm{CO_2}]} \times \frac{5.47\times10^{11}\,\mathrm{cm^3\,ppm}}{\mathrm{kg}_{\mathrm{CO_2}}}$	$\frac{\partial N}{\partial[\mathrm{CO_2}]} \times \frac{5.47\times10^{11}\,\mathrm{cm^3\,ppm}}{\mathrm{kg}_{\mathrm{CO_2}}}$	$\frac{\partial N}{\partial[\mathrm{CO_2}]} \times \frac{5.47\times10^{11}\,\mathrm{cm^3\,ppm}}{\mathrm{kg}_{\mathrm{CO_2}}}$
$\mathrm{EF}[1/\mathrm{kg}_{\mathrm{fuel}}] =$	$\mathrm{EF}[1/\mathrm{kg}_{\mathrm{CO_2}}] \times \frac{3.16\,\mathrm{kg}_{\mathrm{CO_2}}}{\mathrm{kg}_{\mathrm{fuel}}}$	$\mathrm{EF}[1/\mathrm{kg}_{\mathrm{CO_2}}] \times \frac{3.04\,\mathrm{kg}_{\mathrm{CO_2}}}{\mathrm{kg}_{\mathrm{fuel}}}$	$\mathrm{EF}[1/\mathrm{kg}_{\mathrm{CO_2}}] \times \frac{3.16\,\mathrm{kg}_{\mathrm{CO_2}}}{\mathrm{kg}_{\mathrm{fuel}}}$
$\mathrm{EF}[1/\mathrm{km}] =$	$\mathrm{EF}[1/\mathrm{kg}_{\mathrm{fuel}}] \times \frac{6.11}{100\,\mathrm{km}} \times \frac{820\,\mathrm{kg}}{\mathrm{m^3}}$	$\mathrm{EF}[1/\mathrm{kg}_{\mathrm{fuel}}] \times \frac{6.51}{100\,\mathrm{km}} \times \frac{748\,\mathrm{kg}}{\mathrm{m^3}}$	
$\mathrm{EF}[1/\mathrm{kWh}] =$			$\frac{\mathrm{EF}[1/\mathrm{kg}_{\mathrm{fuel}}]}{0.045\times12\,\mathrm{kWh}/\mathrm{kg}_{\mathrm{fuel}}}$

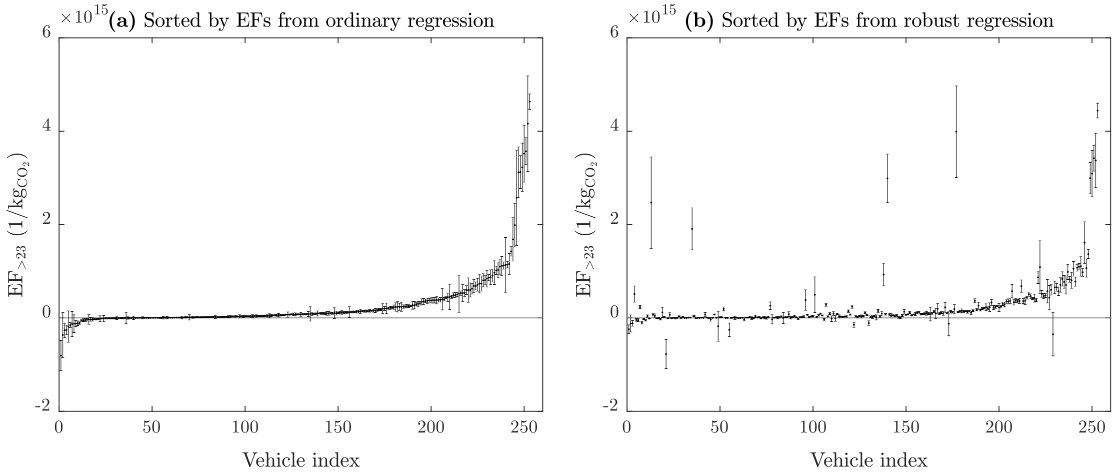

Figure S2: Emission factors of particles larger than 23 nm ($\mathrm{EF}_{>23}$) of all vehicles, obtained using ordinary regression, instead of robust regression as in Fig. 3. Whereas the EF data shown in the both subplots are equal (from ordinary regression), their sorting is different: the vehicles are sorted according to their emission factors obtained (a) using ordinary regression and (b) using robust regression, i.e., the indices in (b) are equal to the ones in Fig. 3 but the EF data not. The error bars denote the standard errors of the slopes in the regressions.

Table S5: Particle number emission factors (EFs) of vehicles with different emission levels. The EF data are shown as medians (with the standard deviations, σ) and means for the vehicle categories (see the footnote for the arrangement of the values). The units of EFs for light-duty (LD) vehicles are 10^{13}/km and for heavy-duty (HD) vehicles 10^{13}/kWh. The number of vehicles in each vehicle category is represented by n. The data in the upper part of the table are presented also graphically in Fig. 5. The lower part presents the data for the particle size bins.

Vehicle category	n	$EF_{>1.3}$	$EF_{>2.5}$	$EF_{>10}$	$EF_{>23}$
LD diesel pre-Euro 4	19	**20.2** (8.40, 79.4); 74.5	**18.0** (8.40, 75.0); 59.6	**10.6** (3.86, 20.2); 15.3	**6.58** (1.75, 17.2); 9.92
LD diesel Euro 4	27	**13.1** (1.57, 45.2); 34.3	**13.1** (1.57, 28.2); 29.3	**8.45** (1.43, 17.4); 9.61	**6.22** (0.591, 15.1); 7.77
LD diesel Euro 5	39	**5.03** (1.12, 32.0); 48.0	**4.66** (0.570, 32.0); 40.0	**1.95** (0.209, 4.36); 5.60	**0.725** (0.0749, 2.58); 2.17
LD diesel Euro 6	35	**3.47** (0.0950, 23.6); 173	**2.59** (0.0950, 21.4); 63.1	**0.615** (0.0548, 5.07); 3.06	**0.312** (0, 2.09); 1.02
LD gasoline pre-Euro 4	27	**12.9** (4.66, 40.3); 40.3	**10.6** (2.68, 30.6); 25.5	**2.58** (0.493, 10.7); 7.71	**0.787** (0.191, 5.13); 4.21
LD gasoline Euro 4	14	**2.10** (0.216, 18.6); 57.7	**0.606** (0.216, 10.2); 43.1	**0.370** (0.136, 5.16); 7.83	**0.216** (0.113, 3.77); 1.51
LD gasoline Euro 5	21	**2.08** (0.234, 20.9); 9.21	**2.08** (0.102, 20.9); 8.73	**0.594** (0.102, 2.37); 2.41	**0.374** (0.0890, 1.27); 1.12
LD gasoline Euro 6	18	**2.15** (0.382, 32.6); 9.65	**1.75** (0.309, 32.6); 9.41	**0.484** (0.177, 2.83); 1.90	**0.268** (0, 0.844); 0.684
HD diesel pre-Euro 4	4	**228** (24.0, 1220); 379	**228** (24.0, 775); 267	**204** (24.0, 225); 122	**32.1** (22.3, 156); 58.8
HD diesel Euro 4	5	**13.9** (0, 465); 161	**13.9** (0, 465); 161	**10.9** (0, 295); 82.7	**8.84** (0, 274); 64.5
HD diesel Euro 5	6	**96.2** (12.5, 482); 130	**49.3** (0, 407); 98.1	**7.36** (0, 97.5); 21.3	**4.66** (0, 97.5); 19.6
HD diesel Euro 6	13	**13.6** (0.0402, 99.6); 35.3	**12.3** (0.0402, 99.6); 35.2	**0.837** (0.0402, 8.76); 5.64	**0.561** (0, 3.85); 2.45

Vehicle category	n	$EF_{1.3-2.5}$	$EF_{2.5-10}$	EF_{10-23}
LD diesel pre-Euro 4	19	**0** (0, 10.3); 15.0	**2.86** (0, 60.1); 44.3	**3.50** (0.528, 6.79); 5.35
LD diesel Euro 4	27	**0** (0, 12.9); 5.01	**1.47** (0, 18.7); 19.7	**1.07** (0, 3.48); 1.84
LD diesel Euro 5	39	**0** (0, 1.34); 7.94	**1.77** (0, 23.1); 34.4	**0.395** (0, 1.98); 3.43
LD diesel Euro 6	35	**0** (0, 1.77); 110	**0.723** (0, 18.4); 60.0	**0.332** (0, 2.53); 2.04
LD gasoline pre-Euro 4	27	**0** (0, 5.36); 14.8	**6.66** (0.0160, 19.3); 17.8	**0.600** (0, 3.24); 3.51
LD gasoline Euro 4	14	**0** (0, 12.3); 14.6	**0.394** (0, 9.37); 35.3	**0.193** (0, 0.715); 6.32
LD gasoline Euro 5	21	**0** (0, 0.730); 0.488	**1.48** (0, 14.2); 6.32	**0.289** (0, 1.89); 1.29
LD gasoline Euro 6	18	**0** (0, 0.264); 0.239	**0.804** (0, 19.6); 7.51	**0.177** (0.0156, 1.95); 1.22
HD diesel pre-Euro 4	4	**0** (0, 446); 111	**24.1** (0, 551); 146	**48.8** (1.71, 193); 62.7
HD diesel Euro 4	5	**0** (0, 0); 0	**3.02** (0, 358); 78.4	**2.05** (0, 68.2); 18.2
HD diesel Euro 5	6	**46.9** (0, 75.5); 31.6	**11.8** (0, 401); 76.8	**1.15** (0, 4.41); 1.65
HD diesel Euro 6	13	**0** (0, 0); 0.102	**7.68** (0, 60.8); 29.5	**0.309** (0, 4.91); 3.19

Median (Median$-\sigma$, Median$+\sigma$); Mean

Table S6: Mean particle number EFs compared to other studies. The units are $10^{13}/kg_{CO_2}$. Other studies report the EFs in the unit of $1/kg_{fuel}$ but they are converted here for comparing purposes. Cut-off diameters in the other studies differing from the ones in this study are marked with the footnotes.

Study	Type	Year	$EF_{>1.3}$	$EF_{>2.5}$	$EF_{>10}$	$EF_{>23}$
Hietikko et al.[S16]	Urban street canyon	2017	[a]130	[c]100		
Lintusaari et al.[S17]	Urban street canyon	2018	[b]110	[c]41	18	5.4
This study	Highway	2020	380	230	44	25
Ban-Weiss et al.[S18]	Highway tunnel, HD	2006		[c]150		
This study	Highway, HD	2020	210	180	67	43
Ježek et al.[S19]	Highway, LD diesel	2011		[d]140		
This study	Highway, LD diesel	2020	540	300	46	27
Ježek et al.[S19]	Highway, LD gasoline	2011		[d]62		
This study	Highway, LD gasoline	2020	190	140	34	14

[a] ~1 nm, [b] 1.4 nm, [c] 3 nm, [d] 5.6 nm

References

(S1) Gormley, P. G.; Kennedy, M. Diffusion from a Stream Flowing through a Cylindrical Tube. *P. Roy. Irish Acad. A* **1948**, *52*, 163–169.

(S2) Brockmann, J. E. In *Aerosol Measurement: Principles, Techniques, and Applications*, 2nd ed.; Baron, P. A., Willeke, K., Eds.; John Wiley & Sons: Hoboken, USA, 2005; pp 143–195.

(S3) Hangal, S.; Willeke, K. Overall efficiency of tubular inlets sampling at 0–90 degrees from horizontal aerosol flows. *Atmos. Environ. A-Gen.* **1990**, *24*, 2379–2386.

(S4) Olin, M.; Dal Maso, M. CFD modeling the diffusional losses of nanocluster-sized particles and condensing vapors in 90° bends of circular tubes. *J. Aerosol Sci.* **2020**, *150*, 105618.

(S5) Collins, A. Ultrafine Particle Loss in Aerosol Diluters. Master of Science thesis, University of Minnesota, 2010.

(S6) Herndon, S. C.; Shorter, J. H.; Zahniser, M. S.; Nelson, D. D.; Jayne, J.; Brown, R. C.; Miake-Lye, R. C.; Waitz, I.; Silva, P.; Lanni, T.; Demerjian, K.; Kolb, C. E. NO and NO2 Emission Ratios Measured from In-Use Commercial Aircraft during Taxi and Takeoff. *Environ. Sci. Technol.* **2004**, *38*, 6078–6084.

(S7) Jayaratne, E.; Morawska, L.; Ristovski, Z.; Johnson, G. The use of carbon dioxide as a tracer in the determination of particle number emissions from heavy-duty diesel vehicles. *Atmos. Environ.* **2005**, *39*, 6812–6821.

(S8) Zavala, M.; Herndon, S. C.; Slott, R. S.; Dunlea, E. J.; Marr, L. C.; Shorter, J. H.; Zahniser, M.; Knighton, W. B.; Rogers, T. M.; Kolb, C. E.; Molina, L. T.; Molina, M. J. Characterization of on-road vehicle emissions in the Mexico City Metropolitan Area

using a mobile laboratory in chase and fleet average measurement modes during the MCMA-2003 field campaign. *Atmos. Chem. Phys.* **2006**, *6*, 5129–5142.

(S9) Wihersaari, H.; Pirjola, L.; Karjalainen, P.; Saukko, E.; Kuuluvainen, H.; Kulmala, K.; Keskinen, J.; Rönkkö, T. Particulate emissions of a modern diesel passenger car under laboratory and real-world transient driving conditions. *Environ. Pollut.* **2020**, *265*, 114948.

(S10) Canagaratna, M. R.; Jayne, J. T.; Ghertner, D. A.; Herndon, S.; Shi, Q.; Jimenez, J. L.; Silva, P. J.; Williams, P.; Lanni, T.; Drewnick, F.; Demerjian, K. L.; Kolb, C. E.; Worsnop, D. R. Chase Studies of Particulate Emissions from in-use New York City Vehicles. *Aerosol Sci. Tech.* **2004**, *38*, 555–573.

(S11) Ježek, I.; Drinovec, L.; Ferrero, L.; Carriero, M.; Močnik, G. Determination of car on-road black carbon and particle number emission factors and comparison between mobile and stationary measurements. *Atmos. Meas. Tech.* **2015**, *8*, 43–55.

(S12) Hansen, A. D. A.; Rosen, H. Individual Measurements of the Emission Factor of Aerosol Black Carbon in Automobile Plumes. *J. Air Waste Manage.* **1990**, *40*, 1654–1657.

(S13) ABC ABC-asemilla myytävien polttonesteiden tuotetiedot. 2022; last access: 16 September 2022, available at: https://old.abcasemat.fi/fi/polttoaineet/tuotetiedot.

(S14) Alakangas, E.; Hurskainen, M.; Laatikainen-Luntama, J.; Korhonen, J. *Properties of fuels used in Finland*; VTT Technology 258; VTT Technical Research Centre of Finland: Finland, 2016.

(S15) Stanton, D. W. Systematic Development of Highly Efficient and Clean Engines to Meet Future Commercial Vehicle Greenhouse Gas Regulations. *SAE Int. J. Engines* **2013**, *6*, 1395–1480.

(S16) Hietikko, R.; Kuuluvainen, H.; Harrison, R. M.; Portin, H.; Timonen, H.; Niemi, J. V.; Rönkkö, T. Diurnal variation of nanocluster aerosol concentrations and emission factors in a street canyon. *Atmos. Environ.* **2018**, *189*, 98–106.

(S17) Lintusaari, H.; Kuuluvainen, H.; Vanhanen, J.; Salo, L.; Portin, H.; Järvinen, A.; Juuti, P.; Hietikko, R.; Teinilä, K.; Timonen, H.; Niemi, J. V.; Rönkkö, T. Sub-23 nm particles dominate non-volatile particle number emissions of road traffic. Manuscript submitted to Environ. Sci. Technol.

(S18) Ban-Weiss, G. A.; Lunden, M. M.; Kirchstetter, T. W.; Harley, R. A. Measurement of Black Carbon and Particle Number Emission Factors from Individual Heavy-Duty Trucks. *Environ. Sci. Technol.* **2009**, *43*, 1419–1424.

(S19) Ježek, I.; Katrašnik, T.; Westerdahl, D.; Močnik, G. Black carbon, particle number concentration and nitrogen oxide emission factors of random in-use vehicles measured with the on-road chasing method. *Atmos. Chem. Phys.* **2015**, *15*, 11011–11026.
